# Supplementary material for: Recovery from spindle checkpoint-mediated arrest requires a novel Dnt1-dependent APC/C activation mechanism
Source: PLoS Genet. 2022 Sep 15;18(9):e1010397. doi: 10.1371/journal.pgen.1010397 (PMC9514617; doi:10.1371/journal.pgen.1010397)
Supplement: S1 Fig — (PDF) [file pgen.1010397.s001.pdf]

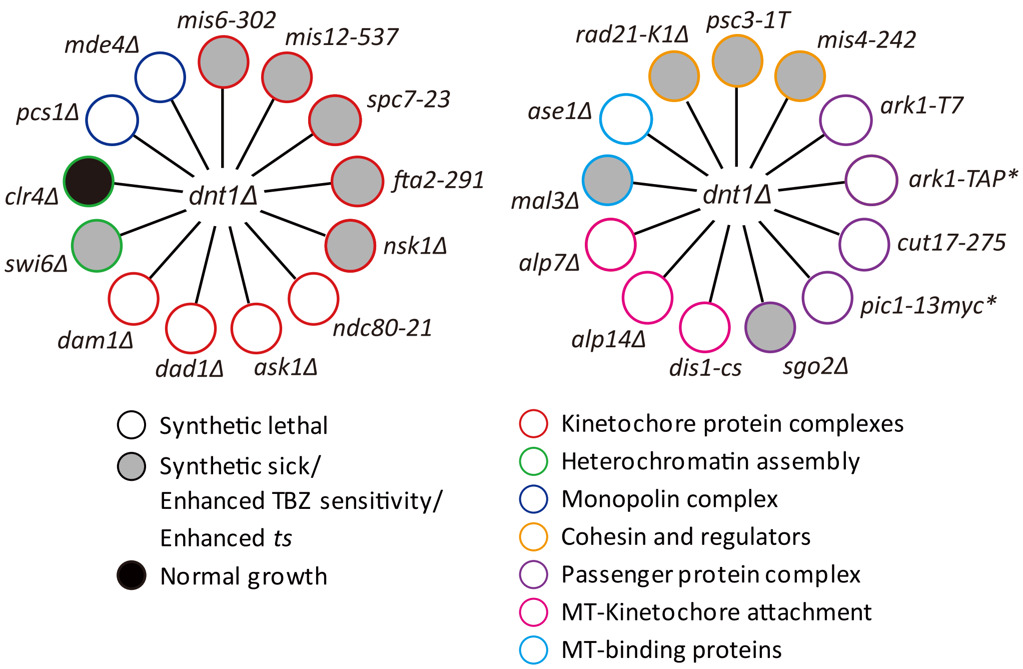
mis6-302
mis12-537
mde4Δ
spc7-23
pcs1Δ
clr4Δ
dnt1Δ
fta2-291
swi6Δ
nsk1Δ
dam1Δ
ndc80-21
dad1Δ
ask1Δ
rad21-K1Δ
psc3-1T
mis4-242
ase1Δ
ark1-T7
mal3Δ
dnt1Δ
ark1-TAP*
cut17-275
alp7Δ
pic1-13myc*
alp14Δ
dis1-cs
sgo2Δ
Synthetic lethal
Synthetic sick/
Enhanced TBZ sensitivity/
Enhanced ts
Normal growth
Kinetochore protein complexes
Heterochromatin assembly
Monopolin complex
Cohesin and regulators
Passenger protein complex
MT-Kinetochore attachment
MT-binding proteins

S1 Fig. Summary of synthetic lethality between *dnt1Δ* and mutants with defective chromosome segregation.

Profiles of synthetic genetic interactions between *dnt1Δ* and indicated yeast mutations are depicted and grouped with different colors based on characterized function of each gene. Open and light grey-filled circles denote synthetic lethal and sick interactions, respectively. For double mutants showing synthetic sick interactions, enhanced TBZ sensitivity or enhanced temperature-sensitivity (*ts*), candidates were obtained by standard tetrad dissections after crosses, and then the sick growth phenotype was confirmed by serial dilution spot assays either on YE plates with different concentrations of TBZ at 30 ℃ for non-*ts* mutants or on YE plates at various temperatures for *ts* mutants.
