# Supplementary material for: Recovery from spindle checkpoint-mediated arrest requires a novel Dnt1-dependent APC/C activation mechanism
Source: PLoS Genet. 2022 Sep 15;18(9):e1010397. doi: 10.1371/journal.pgen.1010397 (PMC9514617; doi:10.1371/journal.pgen.1010397)
Supplement: S2 Fig — (PDF) [file pgen.1010397.s002.pdf]

Bai et al., S2 Fig.

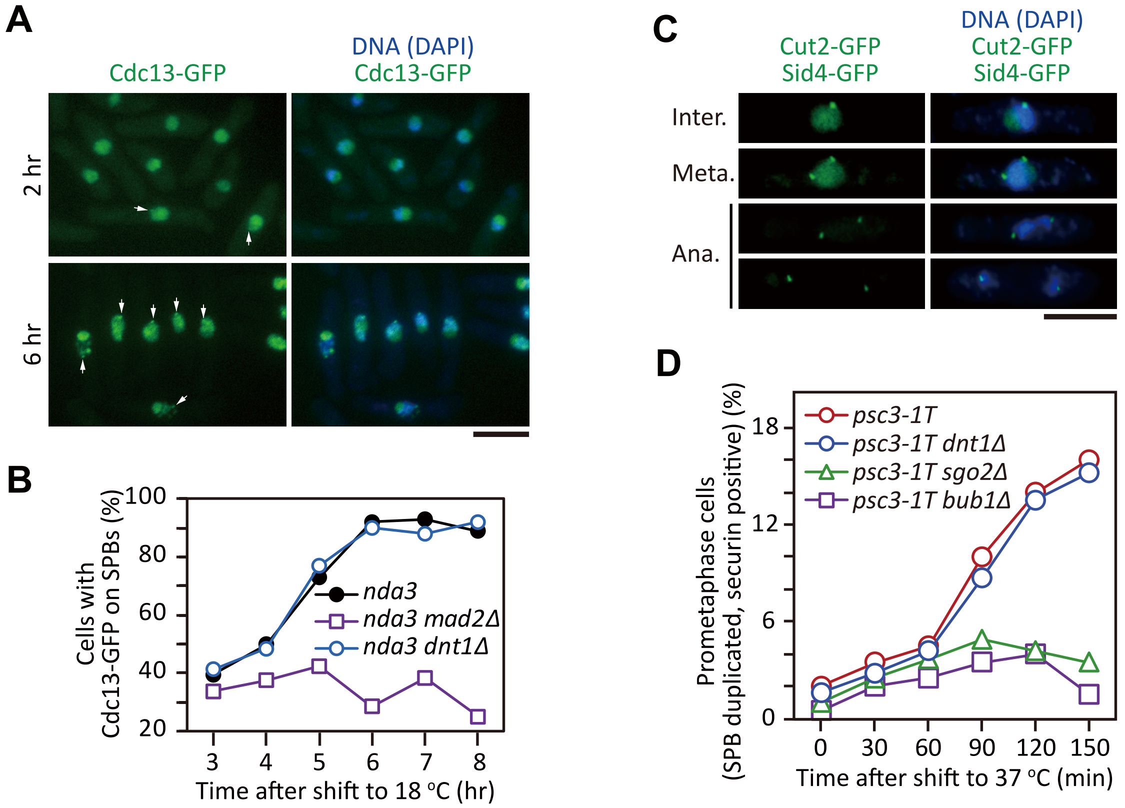

S2 Fig. Dnt1 is dispensable for activating the SAC in the absence of kinetochore-microtubule attachment or tension.

(**A**, **B**) Cyclin B (Cdc13) is accumulated at SPBs in *dnt1Δ* as efficiently as in wild-type in the absence of kinetochore-microtubule attachment. The indicated strains carrying Cdc13-GFP were arrested at S phase by HU at the permissive temperature (30 ℃) for *nda3-KM311*, and released from the arrest to the restrictive temperature (18 ℃). Samples were collected up to 8 hours after release, fixed with methanol and stained with DAPI. Example pictures of *nda3-KM311* cells released after 2 or 6 hours are shown in (**A**). Arrows indicate Cdc13-GFP signals at SPBs. The kinetics of accumulation of Cdc13 at SPBs at each time point was quantified (n > 200 per time point) (**B**). Scale bar, 5 μm.

(**C**, **D**) Dnt1 is not required for activating the spindle checkpoint in the absence of tension. The indicated strains carrying Cut2-GFP and Sid4-GFP were arrested at S phase by HU at 25 ℃ and released to 37 ℃. Examples of Cut2-GFP and Sid4-GFP images at interphase (Inter.), metaphase (Meta.) and anaphase (Ana.) are shown in (**C**). Prometaphase (SPB duplicated and Cut2-positive) cells were counted at each time point (n > 200) (**D**). Note that Cut2-GFP, but not Sid4-GFP at SPBs, disappears from the anaphase cells. Scale bar, 5 μm.
