# Supplementary material for: Recovery from spindle checkpoint-mediated arrest requires a novel Dnt1-dependent APC/C activation mechanism
Source: PLoS Genet. 2022 Sep 15;18(9):e1010397. doi: 10.1371/journal.pgen.1010397 (PMC9514617; doi:10.1371/journal.pgen.1010397)
Supplement: S3 Fig — (PDF) [file pgen.1010397.s003.pdf]

Bai et al., S3 Fig.

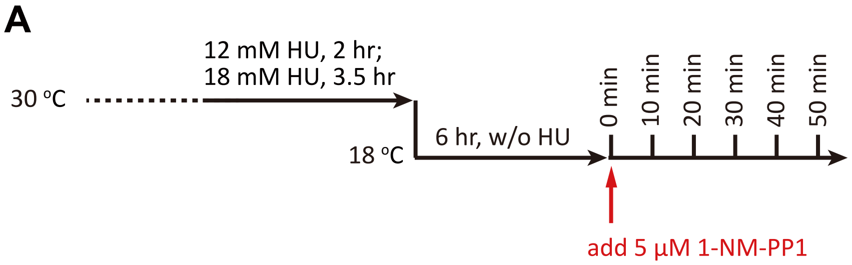

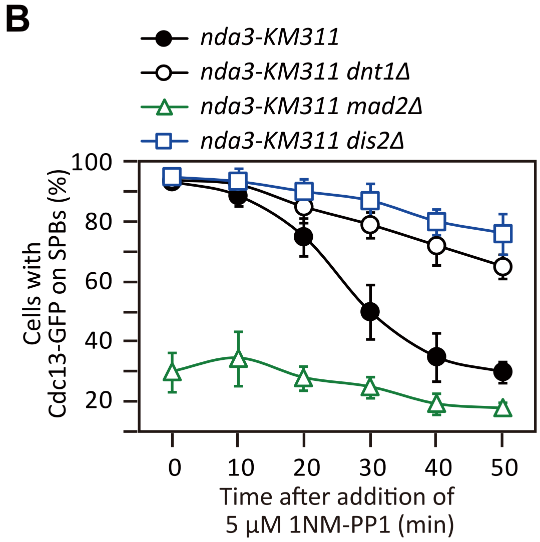

S3 Fig. Related to Fig 2C.

Dnt1 is required to efficiently silence the spindle checkpoint when Aurora B kinase is inhibited in the absence of microtubules.

(**A**) Schematic depiction of the experiment design for checkpoint-silencing assay in which SAC is inactivated by addition of 1-NM-PP1 to mitotically arrested *nda3-KM311 ark1-as3 cdc13-GFP* cells. Cells were grown at the permissive temperature for *nda3-KM311* (30 ℃) to mid-log phase, synchronized at S phase by adding HU to a final concentration of 12 mM for 2 hours followed by a second dose of HU (6 mM final concentration) for 3.5 hours. HU was washed out and cells were released at the restrictive temperature 18 ℃ for 6 hours to be arrested in mitosis, followed by addition of 5 μM 1-NM-PP1, which selectively inhibits analog-sensitive Ark1 kinase (Ark1-as3). Samples were collected at 10 min intervals and fixed by methanol and stained by DAPI for microscopy.

(**B**) The percentage of cells with Cdc13 on spindle poles was assessed at each time point after addition of 1-NM-PP1. Strains lacking *mad2*$^+$ or *dis2*$^+$ are included as controls, because it has been shown by previous studies that *mad2*Δ is defective in SAC activation and *dis2*Δ is defective in SAC inactivation. Each Cdc13-GFP-assessing experiment was repeated three times. Error bars correspond to standard deviation (SD).
