# Supplementary material for: Recovery from spindle checkpoint-mediated arrest requires a novel Dnt1-dependent APC/C activation mechanism
Source: PLoS Genet. 2022 Sep 15;18(9):e1010397. doi: 10.1371/journal.pgen.1010397 (PMC9514617; doi:10.1371/journal.pgen.1010397)
Supplement: S4 Fig — (PDF) [file pgen.1010397.s004.pdf]

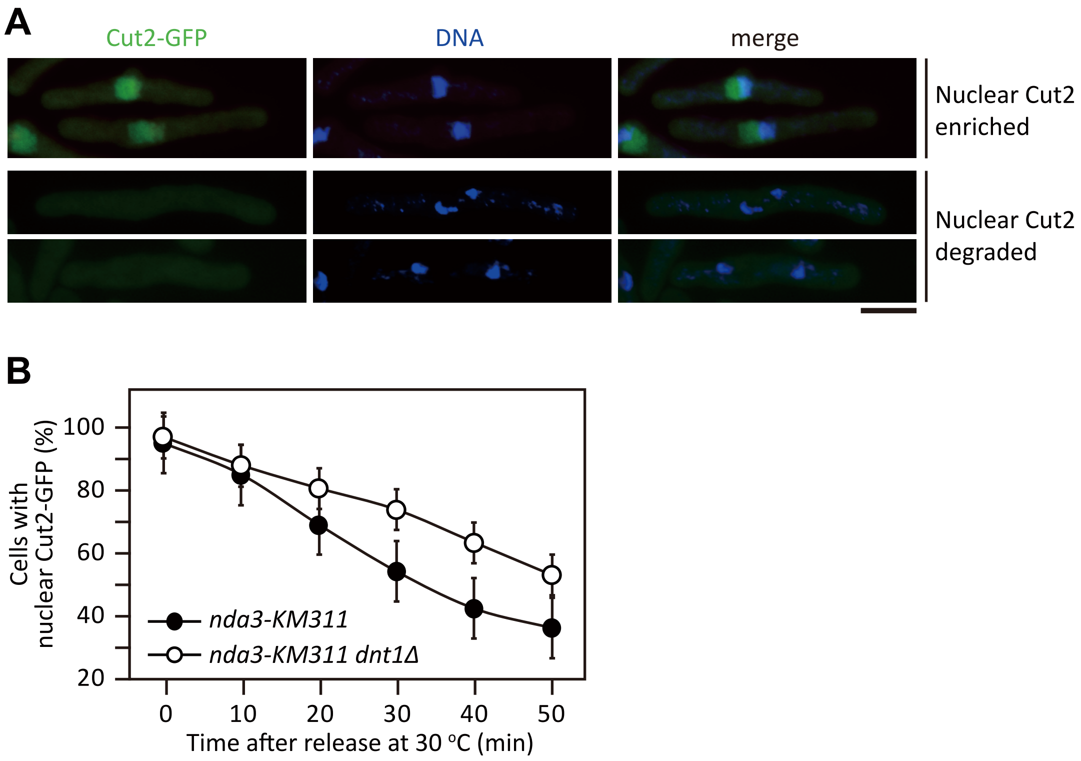
A
Cut2-GFP
DNA
merge
Nuclear Cut2 enriched
Nuclear Cut2 degraded
B
Cells with nuclear Cut2-GFP (%)
100
80
60
40
20
nda3-KM311
nda3-KM311 dnt1Δ
0
10
20
30
40
50
Time after release at 30 °C (min)

S4 Fig. Dnt1 is required for timely degradation of securin (Cut2 in *S. pombe*) upon SAC inactivation.

Cells were grown at the permissive temperature for *nda3-KM311* (30 ℃) to mid-log phase, synchronized at S phase by HU, and then washed and released at the restrictive temperature 18 ℃ for 6 hours and finally shifted back to 30 ℃ as in Fig 2A. Samples were collected at 10 min intervals and fixed for microscopy analysis.

(**A**) Example pictures of cells with nuclear Cut2-GFP signals enriched or degraded are shown. Scale bar, 5 μm.

(**B**) The percentage of cells with nuclear Cut2-GFP signals was assessed at each time point after shift to 30 ℃. Each experiment was repeated three times.

Consistent with the results from Cdc13-GFP (cyclin B) (Fig 2C), deletion of *dnt1*$^{+}$ also causes a delay in securin degradation and thus delay in anaphase initiation and mitotic exit following spindle reassembly and recovery from checkpoint arrest.
