# Supplementary material for: Recovery from spindle checkpoint-mediated arrest requires a novel Dnt1-dependent APC/C activation mechanism
Source: PLoS Genet. 2022 Sep 15;18(9):e1010397. doi: 10.1371/journal.pgen.1010397 (PMC9514617; doi:10.1371/journal.pgen.1010397)
Supplement: S5 Fig — (PDF) [file pgen.1010397.s005.pdf]

Bai et al., S5 Fig.

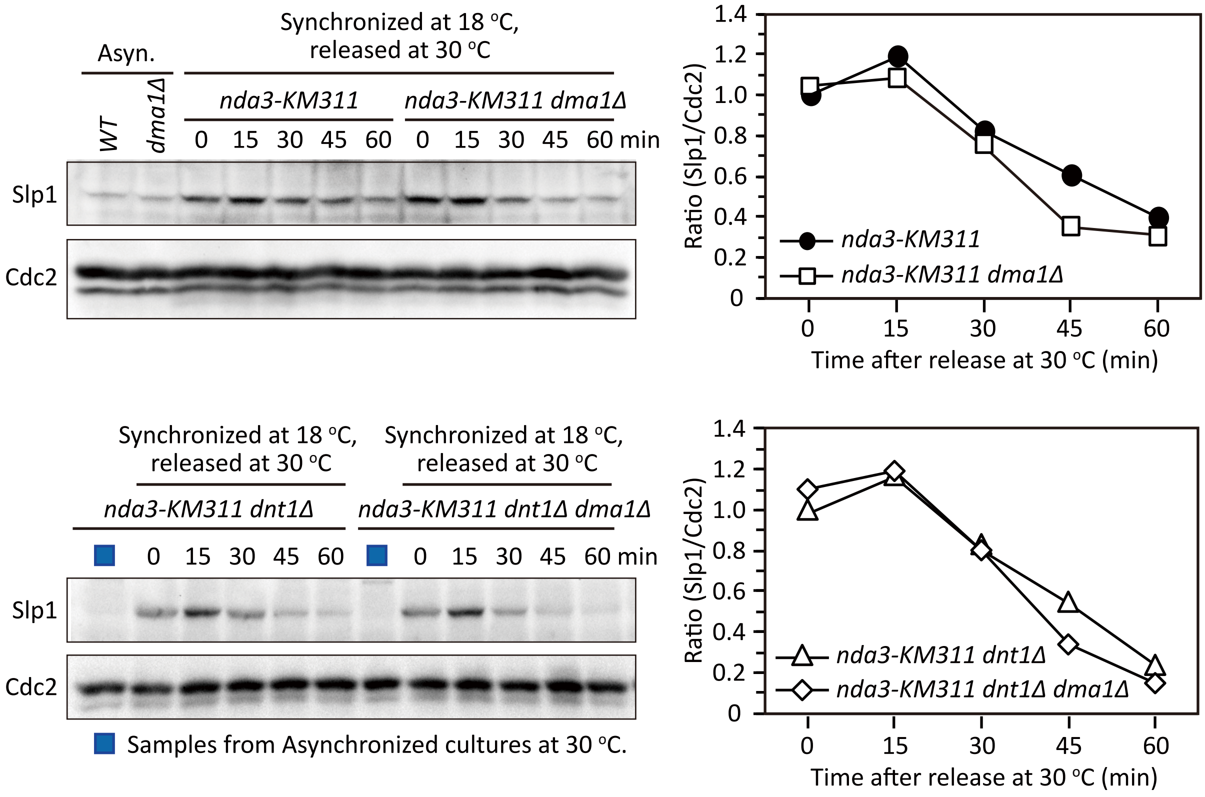

S5 Fig. Dma1 does not affect the abundance of Slp1^{Cdc20} or restore Slp1^{Cdc20} protein level in *dnt1*Δ cells during anaphase after SAC inactivation.

Cells with indicated genotypes were grown at the permissive temperature for *nda3-KM311* (30 ℃) to mid-log phase. Aliquots were withdrawn as asynchronous cultures (Asyn.) and the rest was synchronized at S phase by adding HU to a final concentration of 12 mM for 2 hours followed by a second dose of HU (6 mM final concentration) for 3.5 hours. HU was washed out and cells were released at the restrictive temperature 18 ℃ for 6 hours and finally shifted back to the permissive temperature 30 ℃. (*Left*) Samples were collected at 15 min intervals and subjected to Western blot analyses using anti-Slp1 and anti-Cdc2 antibodies. (*Right*) Slp1^{Cdc20} levels were normalized to those of total Cdc2 at each time point, with the relative ratio between Slp1^{Cdc20} and Cdc2 at 0 min in *nda3-KM311* or *nda3-KM311 dnt1*Δ set as 1.0.

Results are representative of two independent experiments.
