# Supplementary material for: Recovery from spindle checkpoint-mediated arrest requires a novel Dnt1-dependent APC/C activation mechanism
Source: PLoS Genet. 2022 Sep 15;18(9):e1010397. doi: 10.1371/journal.pgen.1010397 (PMC9514617; doi:10.1371/journal.pgen.1010397)
Supplement: S6 Fig — (PDF) [file pgen.1010397.s006.pdf]

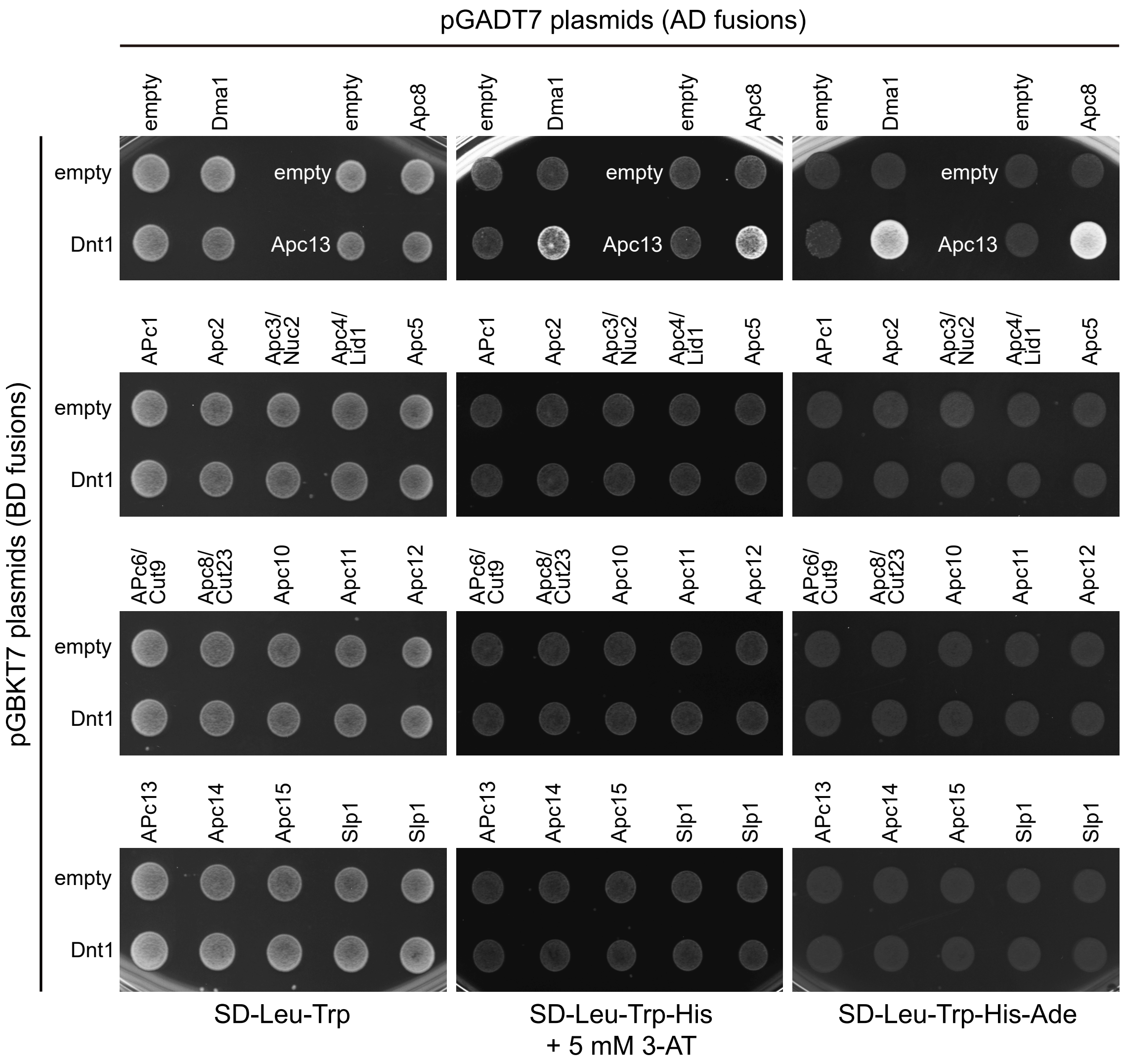
pGADT7 plasmids (AD fusions)
pGBKT7 plasmids (BD fusions)
empty
Dma1
empty
Apc8
empty
Dnt1
empty
Apc13
APc1
Apc2
Apc3/ Nuc2
Apc4/ Lid1
Apc5
APc6/ Cut9
Apc8/ Cut23
Apc10
Apc11
Apc12
APc13
Apc14
Apc15
Slp1
SD-Leu-Trp
SD-Leu-Trp-His + 5 mM 3-AT
SD-Leu-Trp-His-Ade

S6 Fig. Dnt1 does not directly associate with APC/C subunits based on yeast two-hybrid assays.

Dnt1 was expressed from pGBKT7 vector as a Gal4 DNA-binding domain fusion (BD) and paired with each APC/C subunits expressed from pGADT7 vector as Gal4 activating domain fusions (AD). The indicated sets of bait and prey were cotransformed into the tester strain AH109 and their putative interaction was detected on selective (SD-Leu-Trp-His or SD-Leu-Trp-His-Ade) plates. Note that Dnt1 and Dma1 or Apc13 and Apc8 are known interacting pairs, thus served as positive controls.
