# Supplementary material for: Recovery from spindle checkpoint-mediated arrest requires a novel Dnt1-dependent APC/C activation mechanism
Source: PLoS Genet. 2022 Sep 15;18(9):e1010397. doi: 10.1371/journal.pgen.1010397 (PMC9514617; doi:10.1371/journal.pgen.1010397)
Supplement: S7 Fig — (PDF) [file pgen.1010397.s007.pdf]

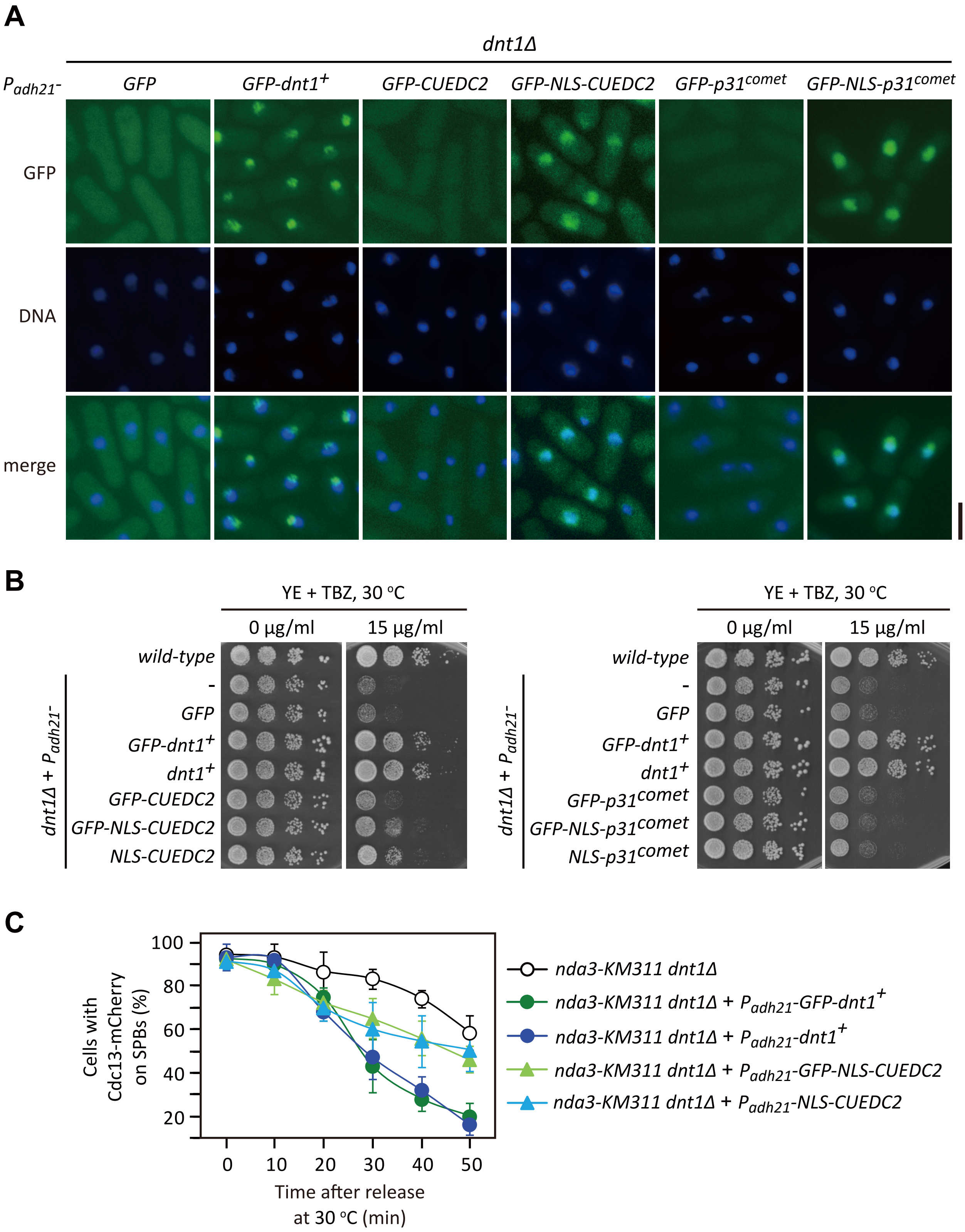
A
dnt1Δ
Padh21-
GFP
GFP-dnt1+
GFP-CUEDC2
GFP-NLS-CUEDC2
GFP-p31comet
GFP-NLS-p31comet
GFP
DNA
merge
B
YE + TBZ, 30 °C
0 µg/ml
15 µg/ml
wild-type
-
GFP
GFP-dnt1+
dnt1+
GFP-CUEDC2
GFP-NLS-CUEDC2
NLS-CUEDC2
dnt1Δ + Padh21-
YE + TBZ, 30 °C
0 µg/ml
15 µg/ml
wild-type
-
GFP
GFP-dnt1+
dnt1+
GFP-p31comet
GFP-NLS-p31comet
NLS-p31comet
dnt1Δ + Padh21-
C
Cells with Cdc13-mCherry on SPBs (%)
100
80
60
40
20
0
10
20
30
40
50
Time after release at 30 °C (min)
nda3-KM311 dnt1Δ
nda3-KM311 dnt1Δ + Padh21-GFP-dnt1+
nda3-KM311 dnt1Δ + Padh21-dnt1+
nda3-KM311 dnt1Δ + Padh21-GFP-NLS-CUEDC2
nda3-KM311 dnt1Δ + Padh21-NLS-CUEDC2

S7 Fig. Human CUEDC2 partially rescues the TBZ sensitivity and spindle checkpoint inactivation defects of *dnt1*Δ cells.

(**A**) Representative images of *dnt1*Δ cells expressing *GFP*, *GFP-dnt1*$^{+}$, *GFP-CUEDC2*, *GFP-2xNLS-CUEDC2*, *GFP-p31*^{comet}, or *GFP-2xNLS-p31*^{comet} under the control of *adh21* promoter (P_{adh21}). Cells from each indicated strain were grown to mid-log phase and then collected and fixed by methanol and stained by DAPI for microscopy. Scale bar, 5 μm.

(**B**) The indicated strains carrying *dnt1*$^{+}$, *CUEDC2*, *2xNLS-CUEDC2*, *p31*^{comet} or *2xNLS- p31*^{comet} with or without GFP tag under the control of *adh21* promoter (P_{adh21}) were grown to mid-log phase and adjusted to equal concentration. 10-fold serial dilutions were spotted and incubated at 30 ℃ for 3-5 days on plates containing the indicated concentrations of TBZ. Note that CUEDC2 without NLS (GFP-CUEDC2) was unable to get into nucleus and could not rescue the TBZ sensitivity of *dnt1*Δ cells.

(**C**) Cells with Cdc13-mCherry and indicated genotypes were grown, mitotically arrested, released and sampled as in Fig 2A. The percentage of cells with Cdc13-mCherry on SPBs was assessed at each time point based on captured microscope images as in Fig 2C. Each experiment was repeated three times.
