# Supplementary material for: Recovery from spindle checkpoint-mediated arrest requires a novel Dnt1-dependent APC/C activation mechanism
Source: PLoS Genet. 2022 Sep 15;18(9):e1010397. doi: 10.1371/journal.pgen.1010397 (PMC9514617; doi:10.1371/journal.pgen.1010397)
Supplement: S8 Fig — (PDF) [file pgen.1010397.s008.pdf]

```
S. pombe Dnt1   MRTTLRLHIIKDGEQDNQFMILFDPSSSISLLKEKVQETYKSLYPFESNINIRNIKNEES  60
Human CUEDC2    ----MEL---------------------------------------------ERI-----  6
                    :.*                                             ..*

S. pombe Dnt1   YDIPNEYLVGEIFPTNSKVIVESFSSPLKKLDGTMINFKEKNI-QHDLDGVENDFATVQS  119
Human CUEDC2    ------------------------------VSAALLAFVQTHLPEADLSGLDEVIFS---  33
                                              :..::: * :..: : **.*::: : :

S. pombe Dnt1   ASNGVHAINGKRTHPDESENPRKLPKKNFVEAIDANSPGFVYRPTSIRDRAYSISSN---  176
Human CUEDC2    YVLGVLEDLGP-----SGPSEENFDMEAFTEMMEAYVPGFAHIPRGTIGDMMQKLSGQLS  88
                   **    *      .. . .::  : *.* ::*  ***.: * .      .  *

S. pombe Dnt1   ----HDNESTLTEGIALKEIESPDKDRKADGIV-----NLSVTQEEDDNHQSFNSSLTPS  227
Human CUEDC2    DARNKENLQPQSSGVQGQVPISPEPLQRPEMLKEETRSSAAAAADTQDEATGAEEELLPG  148
                    ::* .  :.*:  :   **:  :: : :      . :.: : :*:  . :..* *.

S. pombe Dnt1   Q-------PTTYNRANFFSINDASSD----------------SSSDAPLRTLSSPSRLRM  264
Human CUEDC2    VDVLLEVFPTCSVEQAQWVLAKARGDLEEAVQMLVEGKEEGPAAWEGPNQD--LPRRLRG  206
                        **   .   : : .* .*                :: :.* :    * ***

S. pombe Dnt1   --KDNDRKYLVEHSPAALIKESETIDGIDDKSLRSSTREVSVESPNE------DSVNDDS  316
Human CUEDC2    PQKDELKSFILQK--------YMMVDSAEDQKIHRPMA--PKEAPKKLIRYIDNQVVSTK  256
                  **: :.:::::           :*. :*:.::        *:*::      :.* . .

S. pombe Dnt1   SSDVSDEKETEAKHEIRAPAIIVRETSSHPSTAVPSENDTTESENDTLSESSTTSISSSP  376
Human CUEDC2    GERFKDVRNPE-AEEMKATYINL-----------------------------------KP  280
                .. ..* :: *  .*::*  * :                                   .*

S. pombe Dnt1   SENSDTSDDLTKVDSPNKSLVNDNVSAKHDKESENGKSKFPPPSQTLVTTSTISAAGNEP  436
Human CUEDC2    ARK----------------------------------YRFH-------------------  287
                :.:                                   :*

S. pombe Dnt1   SDEIGSENDSDSDSDSDSSVPLSQLQKKSQQRNSVSHEIQNRGTKGSPKEPKAKPSTERP  496
Human CUEDC2    ------------------------------------------------------------  287

S. pombe Dnt1   ETHRTLSYSRLSELSKTFSPEIREPSLTKKTAVSMQESKEEGRSDESSESEESGSSSDES  556
Human CUEDC2    ------------------------------------------------------------  287

S. pombe Dnt1   DNSEKEDRSNPIPVEKRASTVLNTKKKRKAKRNSALAGLAALV           599
Human CUEDC2    -------------------------------------------           287
```

S8 Fig.

Sequence alignment of *S. pombe* Dnt1 and human CUEDC2 by CLUSTAL Omega.

In a 58 amino acid overlap region (marked by red rectangles) (corresponding to Dnt1 (146-196 aa) and CUEDC2 (55-112 aa) respectively), two proteins show 25.9% identity and 55.2% similarity.
