# Supplementary material for: Recovery from spindle checkpoint-mediated arrest requires a novel Dnt1-dependent APC/C activation mechanism
Source: PLoS Genet. 2022 Sep 15;18(9):e1010397. doi: 10.1371/journal.pgen.1010397 (PMC9514617; doi:10.1371/journal.pgen.1010397)
Supplement: S9 Fig — (PDF) [file pgen.1010397.s009.pdf]

Cross: P_{nmt1}-*mad2*$^{+}$*::leu1*$^{+}$ X *cdc20-100*

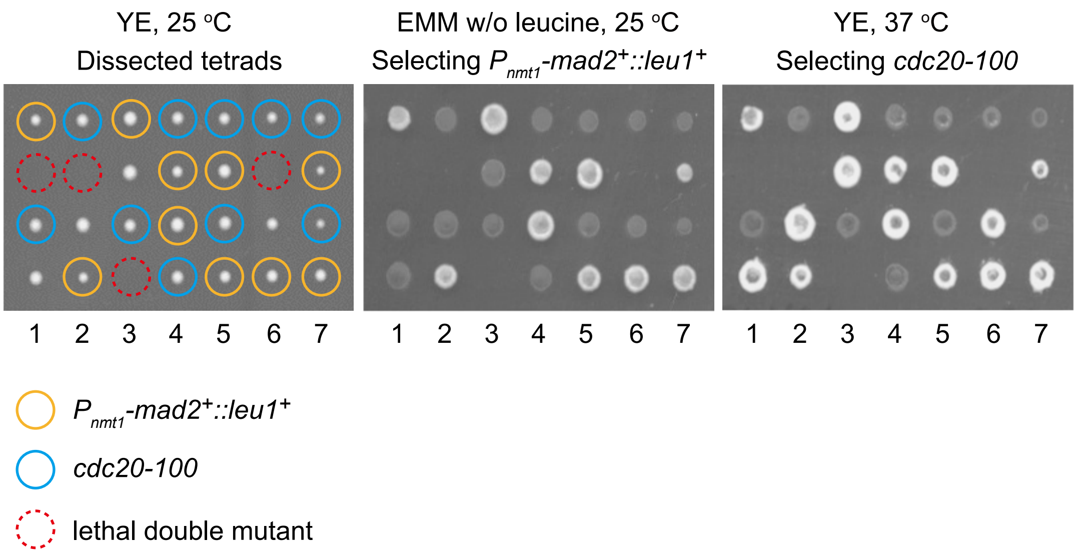

S9 Fig. Related to Fig 6B.

Elevated expression of Mad2 causes strong synthetic lethality in *cdc20-100* mutant background.

Normal-looking 4-spore asci obtained after crosses between the strain carrying P_{nmt1}-*mad2*$^{+}$*::leu1*$^{+}$ and *cdc20-10* mutant strain were dissected using a micromanipulator. The genotypes of colonies formed from germinated spores were deduced after being replicated on selective plates. Dashed red circles indicate all double mutants failed to germinate, indicating double mutants of *cdc20-100* P_{nmt1}-*mad2*$^{+}$*::leu1*$^{+}$ are lethal.
