# Supplementary material for: Recovery from spindle checkpoint-mediated arrest requires a novel Dnt1-dependent APC/C activation mechanism
Source: PLoS Genet. 2022 Sep 15;18(9):e1010397. doi: 10.1371/journal.pgen.1010397 (PMC9514617; doi:10.1371/journal.pgen.1010397)

Figure 2D.

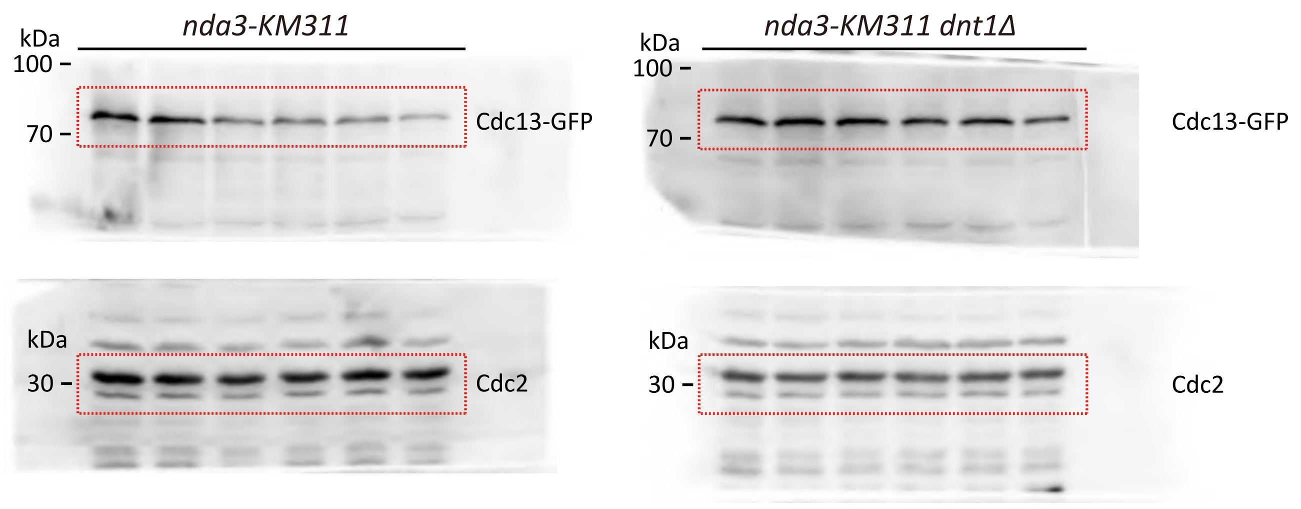

Figure 2F.

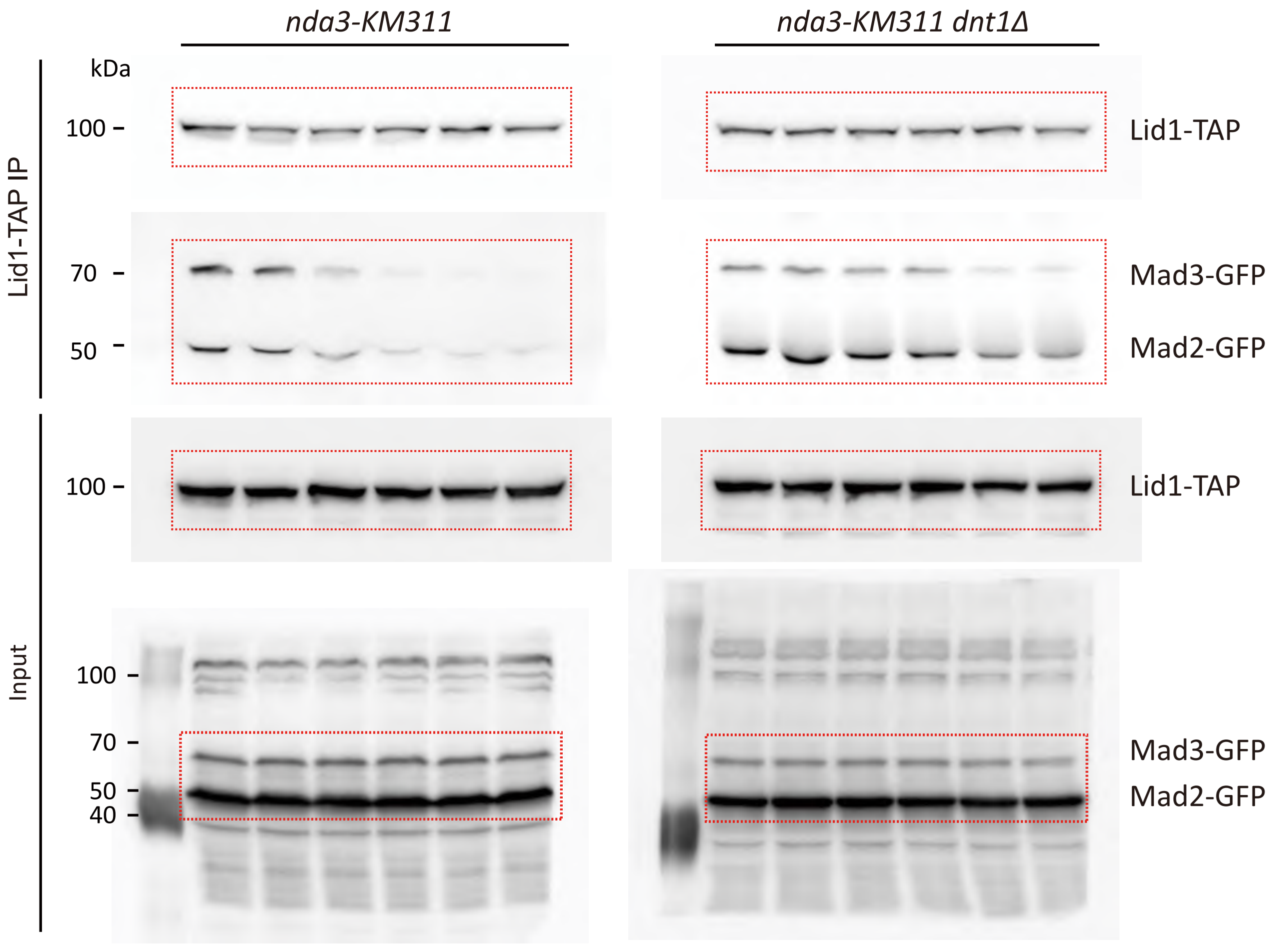

Figure 3B.

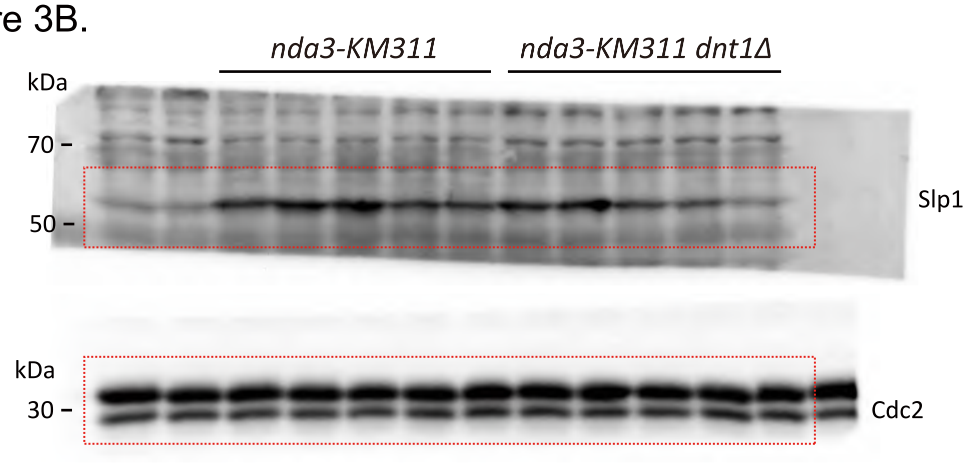

Figure 3D.

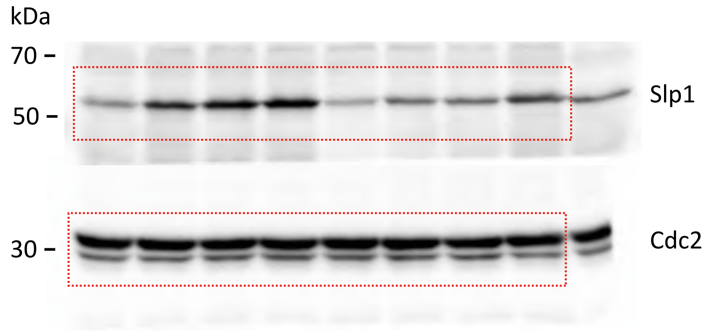

Figure 4A.

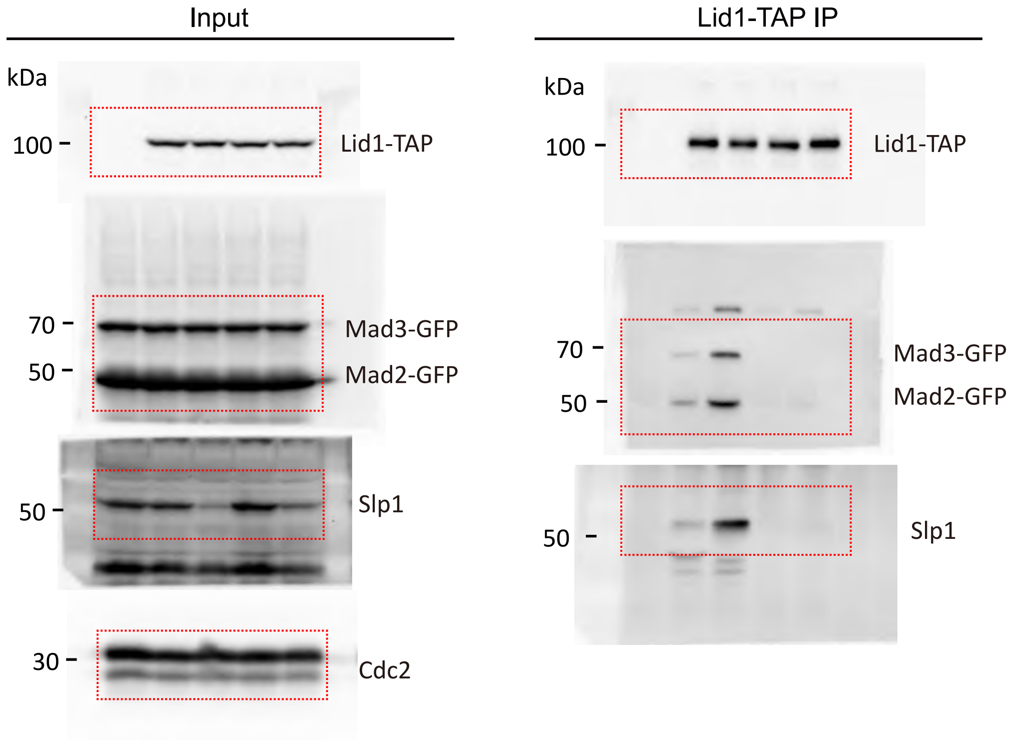

Figure 4B.

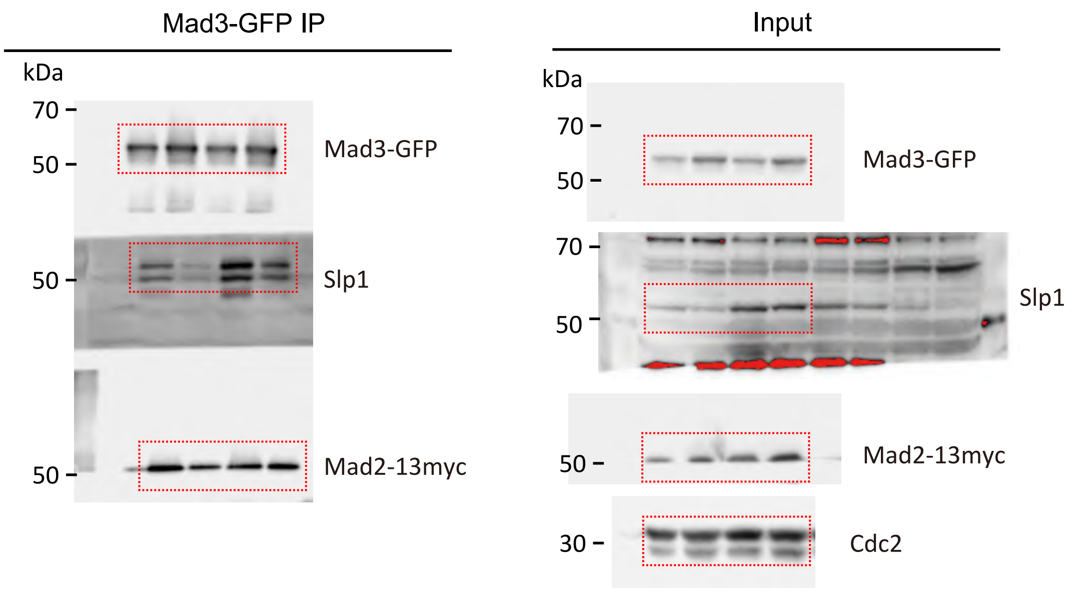

Figure 4C.

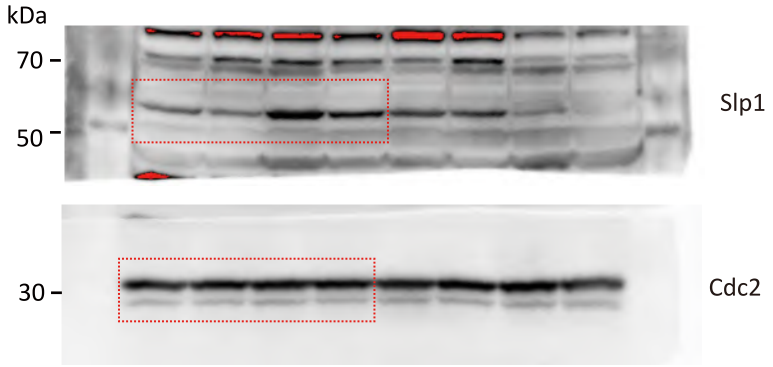

Figure 5A.

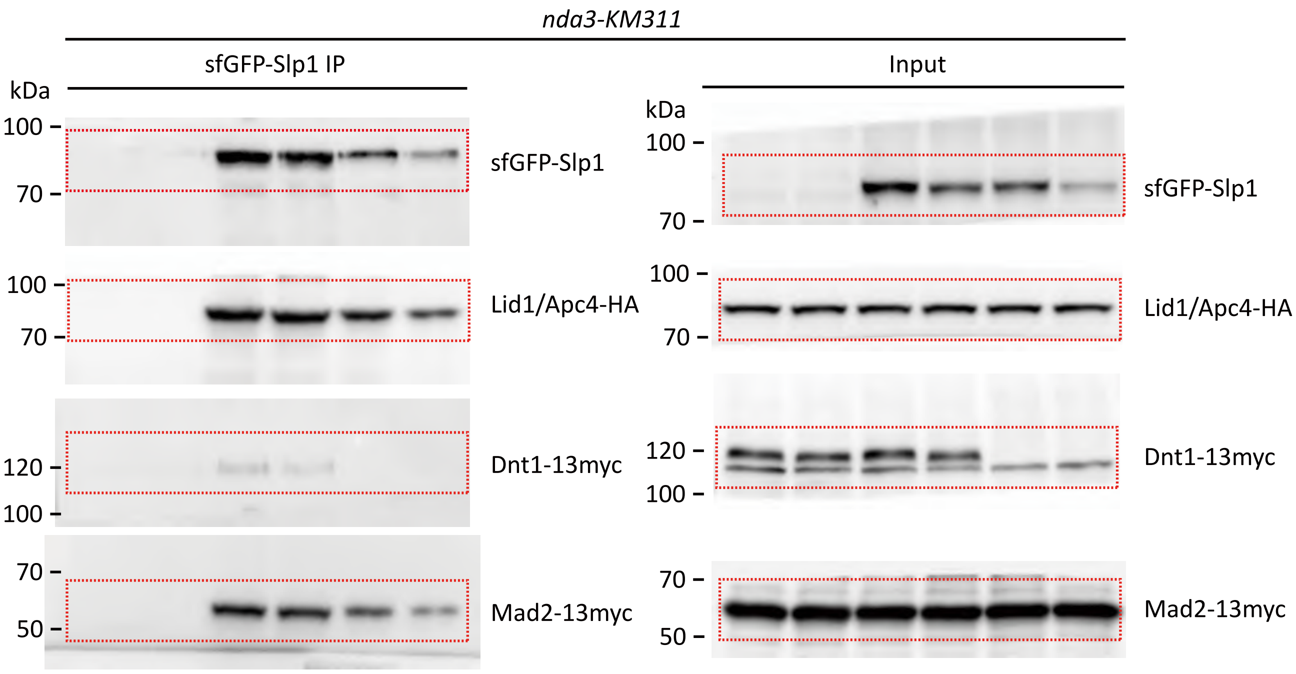

Figure 5B.

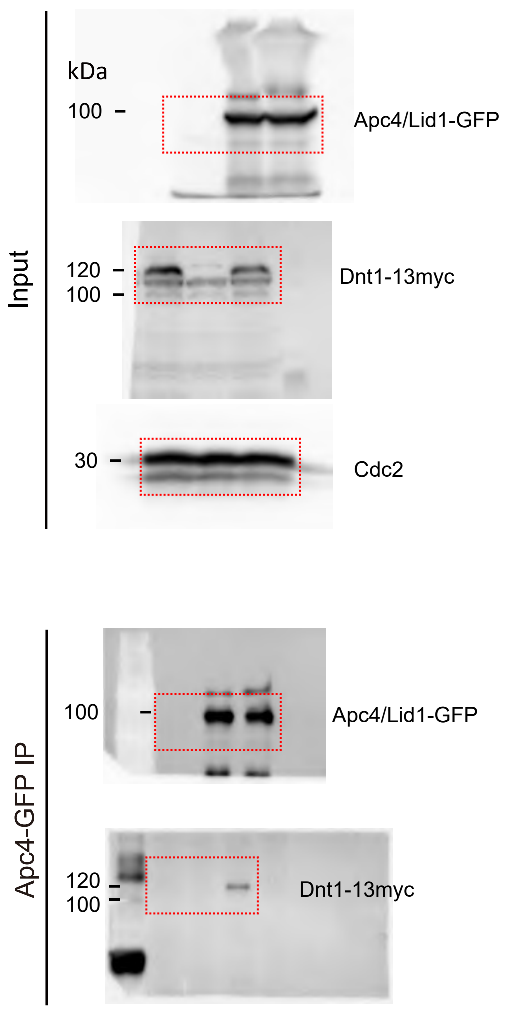

Figure 5C.

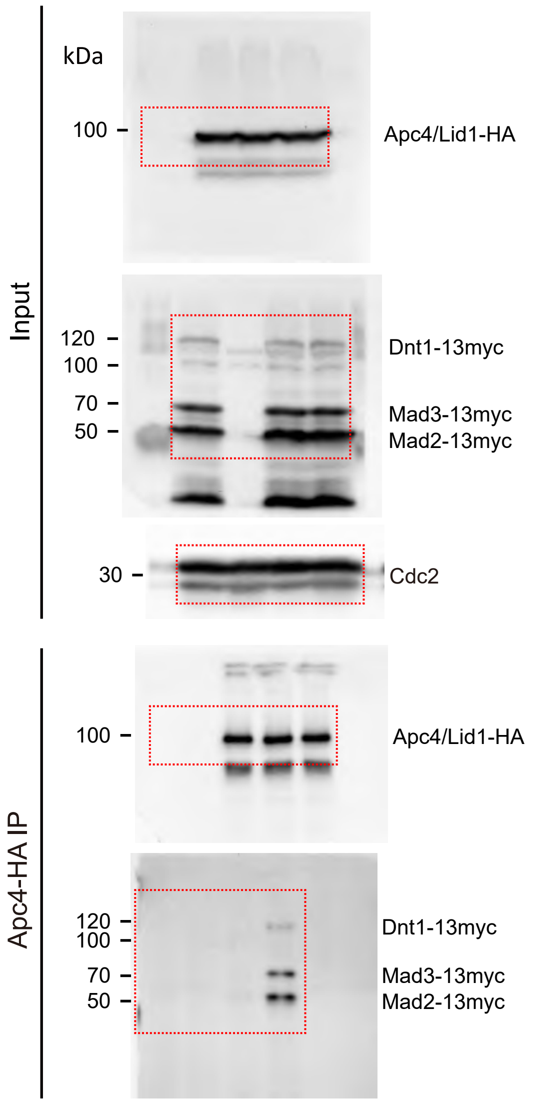

Figure 5D.

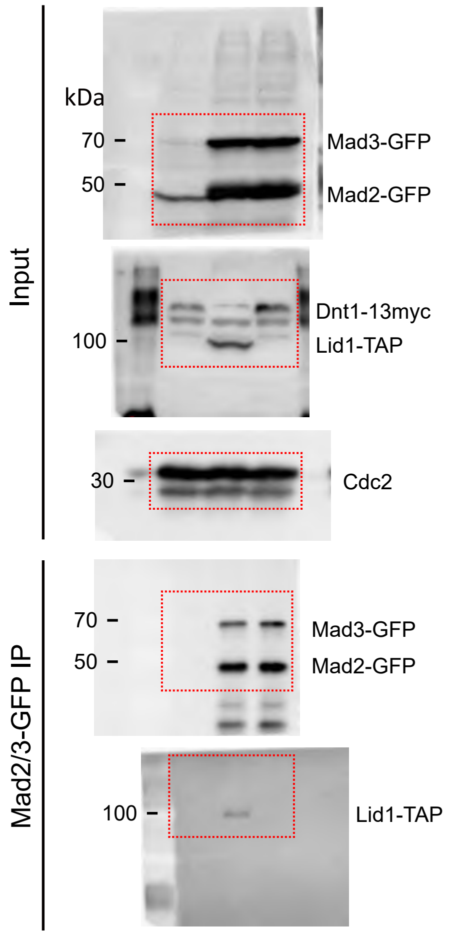

Figure 5E.

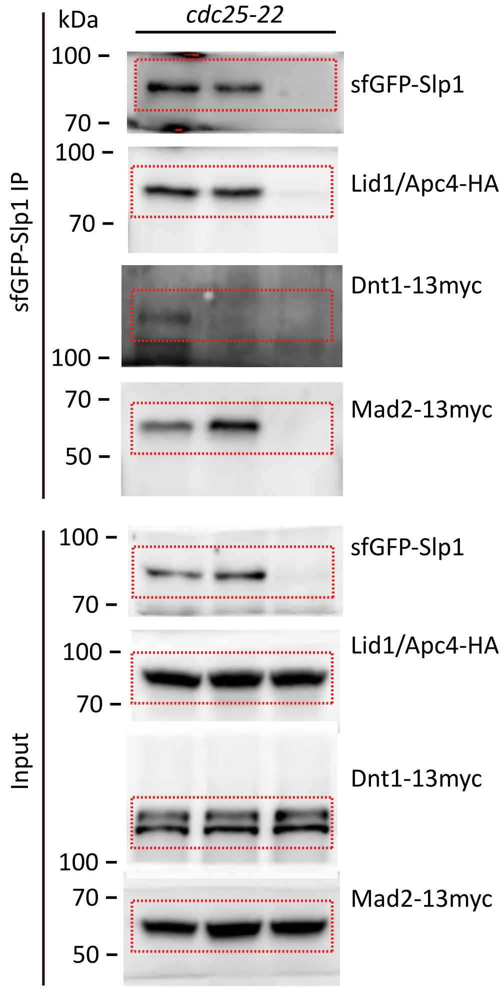

Supplementary Figure 5.

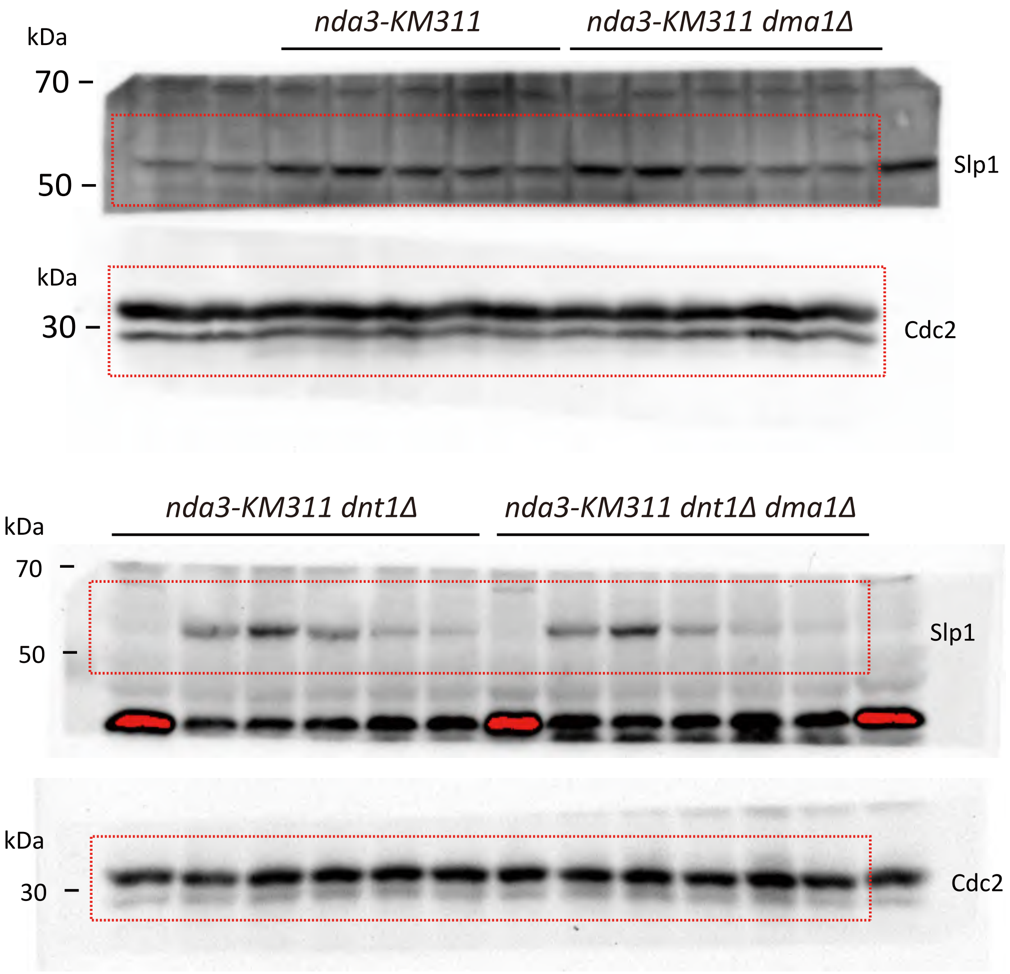

Supplement: S10 Fig — (PDF) [file pgen.1010397.s010.pdf]
