# Supplementary material for: Recovery from spindle checkpoint-mediated arrest requires a novel Dnt1-dependent APC/C activation mechanism
Source: PLoS Genet. 2022 Sep 15;18(9):e1010397. doi: 10.1371/journal.pgen.1010397 (PMC9514617; doi:10.1371/journal.pgen.1010397)
Supplement: S1 Table — (DOC) [file pgen.1010397.s011.doc]

**S1 Table. Yeast strains used in this study.**

| Strain | Genotype | Source |
| --- | --- | --- |
| JY3 | *h^-^ ade6-216 leu1-32 ura4-D18* | Lab stock |
| JY4 | *h^+^ ade6-216 leu1-32 ura4-D18* | Lab stock |
| JY78 | *h^-^ dma1Δ::ura4^+^ leu1-32 ura4-D18 ade6-21x* | [1] |
| JY122 | *h^+^ dnt1Δ::ura4^+^ leu1-32 ura4-D18 ade6-216* | [2] |
| JY2556 | *h^+^ dnt1Δ::ura4^+^ dma1Δ::ura4^+^ leu1-32 ura4-D18 ade6-216* | This study |
| JY1383 | *h? dnt1Δ::ura4^+^* *leu1-32 ura4-D18 ade6-210 Ch16 (ade6-M216)* | This study |
| JY1384 | *h^-^ leu1-32 ura4-D18 ade6-210 Ch16 (ade6-M216)* | [3] |
| JY6692 | *h^+^ mCherry-atb2::hph^R^ sid4-GFP::kan^R^ leu1-32 ura4-D18 ade6-M21X* | Lab stock |
| JY6691 | *h^-^ mCherry-atb2::hph^R^ sid4-GFP::kan^R^ leu1-32 ura4-D18 ade6-M21X* | This study |
| JY6713 | *h? dis2Δ::ura4^+^ mCherry-atb2::hph^R^ sid4-GFP::kan^R^ leu1-32 ura4-D18 ade6?* | This study |
| JY6712 | *h? dnt1Δ::kan^R^ mCherry-atb2::hph^R^ sid4-GFP::kan^R^ leu1-32 ura4-D18 ade6?* | This study |
| JY2453 | *h^-^ ndc80-GFP::kan^R^ sad1-mCherry::nat^R^ leu1-32 ura4-D18* | This study |
| JY2445 | *h^-^ dnt1Δ::ura4^+^ ndc80-GFP::kan^R^ sad1-mCherry::nat^R^ leu1-32 ura4-D18* | This study |
| JY725 | *h^+^ mis6-302 leu1 his2 ura4-D18* | [4] |
| JY2563 | *h^-^ dnt1Δ::ura4^+^ mis6-302 leu1-32 ade6-216* | This study |
| JY724 | *h^+^ mis12-537* | [4] |
| JY774 | *h^+^ mis12-537 ura4-D18 leu1-32* | This study |
| JY775 | *h^+^ mis12-537 ura4-D18 leu1-32* | This study |
| JY2564 | *h^+^ dnt1Δ::ura4^+^ mis12-537 leu1-32 ade6-216 ura4-D18?* | This study |
| JY7347 | *h^+^ spc7-23-GFP::kan^R^::his3^+^ ura4-D18 leu1-32 ade6-210* | [5] |
| JY5833 | *h? dnt1Δ::kan^R^ spc7-23-GFP::kan^R^::his3^+^ ura4-D18 leu1-32 ade6-21x his3-D1?* | This study |
| JY5834 | *h? dnt1Δ::kan^R^ spc7-23-GFP::kan^R^::his3^+^ ura4-D18 leu1-32 ade6-21x his3-D1?* | This study |
| JY7348 | *h^+^ fta2-291::his3^+^ ura4-D18 leu1-32 ade6-210* | [5] |
| JY5835 | *h? dnt1Δ::kan^R^ fta2-291::his3^+^ ura4-D18 leu1-32 ade6-210 his3* | This study |
| JY5836 | *h? dnt1Δ::kan^R^ fta2-291::his3^+^ ura4-D18 leu1-32 ade6-210 his3?* | This study |
| KGY6449 | *h^-^ nsk1Δ::ura4+ ura4-D18 ade6-M21X leu1-32* | [6] |
| JY5825 | *h^+^ dnt1Δ::ura4^+^ nsk1::nsk1-18A-linker-GFP::kan^R^ ura4-D18 ade6-M21X leu1-32* | This study |
| JY5826 | *h^-^ dnt1Δ::ura4^+^ nsk1::nsk1-18A-linker-GFP::kan^R^ ura4-D18 ade6-M21X leu1-32* | This study |
| JY5827 | *h^-^ dnt1Δ::ura4^+^ nsk1::nsk1-18D-linker-GFP::kan^R^ ura4-D18 ade6-M21X leu1-32* | This study |
| JY5828 | *h^+^ dnt1Δ::ura4^+^ nsk1::nsk1-18A-linker-GFP::kan^R^ ura4-D18 ade6-M21X leu1-32* | This study |
| JY5866 | *h^-^ ndc80-21::kan^R^ ura4? leu1?* | [7] |
| JY2511 | *h^-^ ask1Δ::hyg^R^ ura4-D18* | [8] |
| JY2512 | *h^-^ dad1Δ::hyg^R^ leu1-32 ura4-D18* | [9,10] |
| JY2509 | *h^+^ dam1Δ::kan^R^ leu1-32 ura4-D18* | [9,10] |
| JY170 | *h^-^ swi6Δ::ura4^+^ leu1-32 ura4-D18 ade6-21X* | [11] |
| JY171 | *h^+^ dnt1Δ::kan^R^ swi6Δ::ura4^+^ leu1-32 ura4-D18 ade6-21X* | This study |
| JY381 | *h^-^ clr4Δ::kan^R^ leu1-32 ura4-D18 ade6-21X* | [12] |
| JY456 | *h^-^ dnt1Δ::ura4^+^ clr4Δ::kan^R^ leu1-32 ura4-D18 ade6-21X* | This study |
| JY457 | *h? dnt1Δ::ura4^+^ clr4Δ::kan^R^ leu1-32 ura4-D18 ade6-21X* | This study |
| JY3275 | *h^-^ pcs1Δ::kan^R^ leu1- ura4-* *ade6+* | [13] |
| JY3276 | *h^+^ pcs1Δ::kan^R^ leu1- ura4- ade6+* | [14] |
| JY258 | *h^+^ mde4Δ::kanMX lys1 his7 leu1 ura4 ade6-210* | [15] |
| JY259 | *h^-^ mde4Δ::kanMX leu1 ura4 ade6-210* | [15] |
| JY195 | *h^-^ sgo2Δ::ura4^+^ leu1- ura4-D18 ade6-21X* | [16] |
| JY288 | *h^-^ dnt1Δ::kan^R^ sgo2Δ::ura4^+^ leu1-32 ura4-D18 ade6-21X* | This study |
| JY2103 | *h^-^ rad21-K1Δ::ura4^+^ leu1-32 ura4-D18 ade6-21X* | [17] |
| JY2104 | *h? dnt1Δ::kan^R^ rad21-K1Δ::ura4^+^ leu1 ura4-D18 ade6-21X*  *his7* | This study |
| JY315 | *h^-^ psc3-1T-HA::kan^R^ leu1 ura4 ade6-M210* | [16] |
| JY581 | *h^-^ dnt1∆::ura4^+^ psc3-1T-HA-kan^R^ cut2-6GFP[LEU2] leu1 ura4* | This study |
| JY418 | *h^-^ mis4-242* | [4] |
| JY419 | *h^+^ mis4-242* | [4] |
| JY2748 | *h? dnt1Δ::kan^R^ mis4-242 leu1-32? ura4-D18 ade6-210?* | This study |
| JY2749 | *h? dnt1Δ::kan^R^ mis4-242 leu1-32? ura4-D18 ade6-210?* | This study |
| JY570 | *h^-^ ark1-T7::kan^R^ leu1 ura4-D18* | [16] |
| JY571 | *h^-^ ark1-T7::kan^R^ leu1* | [16] |
| JY2092 | *h^+^ ark1-TAP::kan^R^ leu1-32 ura4 ade6?* | Lab stock |
| JY99 | *h^-^ cut17-275 leu1-32 ura4-D18 ade6-216* | [18] |
| JY100 | *h^+^ cut17-275 leu1-32 ura4-D18 ade6-216* | [18] |
| JY133 | *h^-^ pic1-13myc::kan^R^ leu1-32 ura4-D18 ade6-216* | [19] |
| JY153 | *h^-^ pic1-13myc::kan^R^ leu1-32 ura4-D18 ade6-216* | [19] |
| JY229 | *h^+^ dis1Δ::ura4^+^ lys1-131* | [20] |
| JY521 | *h^-^ alp14Δ::kan^R^ leu1-32 ura4-D18* | [21] |
| JY433 | *h^-^ alp7Δ::ura4^+^ leu1 ura4* | [22] |
| JY434 | *h^-^ alp7Δ::kan^R^ leu1 ura4* | [22] |
| JY1965 | *h? mal3Δ::kan^R^ leu1-32 ura4-D18 ade6-M216 his3-D1* | [23] |
| DM3020 | *h? dnt1∆::kan^R^ mal3∆::ura4^+^ leu1-32 ura4-D18 ade6-210* | This study |
| JY2239 | *h^+^ ase1Δ::kan^R^ leu1-32 ura4-D18* | [24] |
| JY228 | *h^+^ nda3-KM311 leu-32 ura4-D18* | Lab stock |
| JY2899 | *h^-^ nda3-KM311 dnt1Δ::ura4^+^ leu1-32 ura4-D18 ade6-21X/216 his3-D1* | This study |
| JY3358 | *h^-^ nda3-KM311 dnt1Δ::ura4^+^ dma1Δ::ura4^+^ alp4-GFP::kan^R^ leu1-32 ura4-D18 ade6-21x?* | This study |
| JY3058 | *h^-^ nda3-KM311 cdc13-117::cdc13-GFP::LEU2 ark1-as3::hyg^R^ ura4-* | [25] |
| JY3092 | *h^+^ nda3-KM311 dnt1Δ::kan^R^ cdc13-117::cdc13-GFP::LEU2 ark1-as3::hyg^R^* | This study |
| JY3093 | *h^-^ nda3-KM311 dnt1Δ::kan^R^ cdc13-117::cdc13-GFP::LEU2 ark1-as3::hyg^R^* | This study |
| JY3094 | *h^+^ nda3-KM311 dnt1Δ::kan^R^ cdc13-117::cdc13-GFP::LEU2 ark1-as3::hyg^R^* | This study |
| JY3600 | *h^-^ nda3-KM311 mad2Δ::ura4^+^ cdc13-117::cdc13-GFP::LEU2 ark1-as3::hyg^R^* | This study |
| JY4510 | *h? nda3-KM311 mad2Δ::ura4^+^ cdc13-117::cdc13-GFP::LEU2 ark1-as3::hyg^R^* | This study |
| JY4511 | *h? nda3-KM311 mad2Δ::ura4^+^ cdc13-117::cdc13-GFP::LEU2 ark1-as3::hyg^R^* | This study |
| JY3602 | *h^-^ nda3-KM311 mad3Δ::ura4^+^ cdc13-117::cdc13-GFP::LEU2 ark1-as3::hyg^R^* | This study |
| JY3603 | *h^+^ nda3-KM311 mad3Δ::ura4^+^ cdc13-117::cdc13-GFP::LEU2 ark1-as3::hyg^R^* | This study |
| JY4514 | *h^-^ nda3-KM311 bub1Δ::ura4^+^ cdc13-117::cdc13-GFP::LEU2 ark1-as3::hyg^R^* | This study |
| JY4515 | *h? nda3-KM311 bub1Δ::ura4^+^ cdc13-117::cdc13-GFP::LEU2 ark1-as3::hyg^R^* | This study |
| JY3060 | *h^-^ nda3-KM311 dis2Δ::ura4^+^ cdc13-117::cdc13-GFP::LEU2 ark1-as3::hyg^R^* | This study |
| JY4512 | *h? nda3-KM311 dnt1Δ::kan^R^ mad2Δ::ura4^+^ cdc13-117::cdc13-GFP::LEU2 ark1-as3::hyg^R^* | This study |
| JY4513 | *h? nda3-KM311 dnt1Δ::kan^R^ mad2Δ::ura4^+^ cdc13-117::cdc13-GFP::LEU2 ark1-as3::hyg^R^* | This study |
| JY3547 | *h^-^ nda3-KM311 dnt1Δ::kan^R^ mad3Δ::ura4^+^ cdc13-117::cdc13-GFP::LEU2 ark1-as3::hyg^R^* | This study |
| JY4545 | *h? nda3-KM311 dnt1Δ::kan^R^ bub1Δ::ura4^+^ cdc13-117::cdc13-GFP::LEU2 ark1-as3::hyg^R^* | This study |
| JY4546 | *h? nda3-KM311 dnt1Δ::kan^R^ bub1Δ::ura4^+^ cdc13-117::cdc13-GFP::LEU2 ark1-as3::hyg^R^* | This study |
| JY5458 | *h^-^ nda3-KM311 cdc13-117::cdc13-GFP::LEU2 apc15Δ::kan^R^ ark1-as3::hyg^R^* | This study |
| JY5459 | *h^+^ nda3-KM311 cdc13-117::cdc13-GFP::LEU2 apc15Δ::kan^R^ ark1-as3::hyg^R^* | This study |
| JY6535 | *h-nda3-KM311 cdc13-117::cdc13-GFP::LEU2 apc15Δ::kan^R^ dnt1Δ::ura4^+^ ark1-as3::hyg^R^* | This study |
| JY6536 | *h^-^ nda3-KM311 cdc13-117::cdc13-GFP::LEU2 apc15Δ::kan^R^ dnt1Δ::ura4^+^ ark1-as3::hyg^R^* | This study |
| JY1475 | *h^+^ psc3-1TS-HA::kan^R^ cut2-GFP::LEU2 sid4-GFP::kan^R^ leu1- ura4- ade6?* | This study |
| JY1479 | *h^-^ psc3-1TS-HA::kan^R^ dnt1Δ::ura4^+^ cut2-GFP::LEU2 sid4-GFP::kan^R^ leu1- ura4- ade6?* | This study |
| JY1566 | *h^+^ psc3-1TS-HA::kan^R^ sgo2Δ::ura4^+^ cut2-GFP::LEU2 sid4-GFP::kan^R^ leu1- ura4- ade6?* | This study |
| JY1605 | *h^+^ psc3-1TS-HA::kan^R^ bub1Δ::ura4^+^ cut2-GFP::LEU2 sid4-GFP::kan^R^ leu1- ura4- ade6?* | This study |
| JY5543 | *h^-^ nda3-KM311 dnt1Δ::kan^R^ cut2-GFP::LEU2 leu1* | This study |
| JY5544 | *h^-^ nda3-KM311 cut2-GFP::LEU2 leu1-* | This study |
| JY4022 | *h? nda3-KM311 lid1-TAP::kan^R^ mad2-GFP::kan^R^ mad3-GFP::his3^+^ leu1-32 ura4-D18 his3-D1 ade6-216* | This study |
| JY3911 | *h? nda3-KM311 dnt1Δ::ura4^+^ lid1-TAP::kan^R^ mad2-GFP::kan^R^ mad3-GFP::his3^+^ his3-D1 leu1-32 ura4-D18 ade6-* | This study |
| JY8872 | *h^+^ nda3-KM311 dma1Δ::kan^R^ lid1-TAP::kan^R^ mad2-GFP::kan^R^ mad3-GFP::his3^+^ leu1-32 ura4-D18 ade6-* | This study |
| JY8874 | *h^+^ nda3-KM311 dnt1Δ::ura4^+^ dma1Δ::kan^R^ lid1-TAP::kan^R^ mad2-GFP::kan^R^ mad3-GFP::his3^+^ leu1-32 ura4-D18 ade6-* | This study |
| JY8875 | *h^+^ nda3-KM311 dnt1Δ::ura4^+^ dma1Δ::kan^R^ lid1-TAP::kan^R^ mad2-GFP::kan^R^ mad3-GFP::his3^+^ leu1-32 ura4-D18 ade6-* | This study |
| JY2750 | *h^+^ P_nmt1_-mad2::leu1^+^ leu1-32 ura4-D18 ade6-210* | Lab stock |
| JY7643 | *h^-^ lys1Δ::P_adh1_-dnt1::hyg^R^ leu1-* | This study |
| JY3509 | *h^-^ cut9-234 leu1* | [26] |
| JY7667 | *h^-^ cut9-234 leu1-32::P_nmt1_-mad2::leu1^+^* | This study |
| JY7501 | *h^-^ cut9-234 lys1Δ::P_adh1_-dnt1::hyg^R^ leu1-* | This study |
| JY7644 | *h^-^ cut9-234 leu1-32::P_nmt1_-mad2::leu1^+^ lys1Δ::P_adh1_-dnt1::hyg^R^* | This study |
| JY3510 | *h^-^ cut20-100 leu1* | [27] |
| JY7502 | *h^-^ cut20-100 lys1Δ::P_adh1_-dnt1::hyg^R^ leu1-* | This study |
| JY7538 | *h? cut20-100 leu1-32::P_nmt1_-mad2::leu1^+^ lys1Δ::P_adh1_-dnt1::hphMX6* | This study |
| JY3512 | *h^-^ cut23-547 leu1* | [26] |
| JY7540 | *h^+^ cut23-547 leu1-32::P_nmt1_-mad2::leu1^+^* | This study |
| JY7503 | *h^-^ cut23-547 lys1Δ::P_adh1_-dnt1::hyg^R^ leu1-* | This study |
| JY7521 | *h^-^ cut23-547 leu1-32::P_nmt1_-mad2::leu1^+^ lys1Δ::P_adh1_-dnt1::hphMX6* | This study |
| JY3508 | *h^-^ nuc2-663 leu1* | [28] |
| JY3554 | *h^+^ dnt1Δ::kan^R^ nuc2-663 ura4- leu1-* | This study |
| JY3509 | *h^-^ cut9-234 leu1* | [26] |
| JY3604 | *h^-^ dnt1Δ::kan^R^ cut9-234 ura4- leu1-* | This study |
| JY3606 | *h^-^ dnt1Δ::kan^R^ cut20-100 ura4- leu1-* | This study |
| JY3610 | *h^-^ dnt1Δ::kan^R^ cut23-547 ura4- leu1-* | This study |
| JY5383 | *h^+^ apc15Δ::kan^R^ ura4-D18 leu1-32 ade6-21X* | [29] |
| JY6578 | *h^+^ dnt1Δ::ura4^+^ apc15Δ::kan^R^ leu1-32 ura4-D18 ade6-21X* | This study |
| JY3511 | *h^-^ slp1-362 ade6-M216 leu1* | [30] |
| JY3608 | *h^-^ dnt1Δ::kan^R^ slp1-362 ura4- leu1-* | This study |
| JY5821 | *h^+^ slp1-mr63* | [31] |
| JY6832 | *h^-^ dnt1Δ::ura4^+^ slp1-mr63* | This study |
| JY7657 | *h^-^ cut20-100 mad2Δ::ura4^+^ leu1-* | This study |
| JY7986 | *h^+^ cut20-100 mad3Δ::ura4^+^ ura4-* | This study |
| JY7585 | *h^+^ cut20-100 dnt1Δ::kan^R^ mad2Δ::ura4^+^ ura4- leu1-* | This study |
| JY7987 | *h^+^ cut20-100 dnt1Δ::kan^R^ mad3Δ::ura4^+^ ura4-* | This study |
| JY7659 | *h^-^ cut23-547 mad2Δ::ura4^+^ leu1-* | This study |
| JY7988 | *h^+^ cut23-547 mad3Δ::ura4^+^ ura4-* | This study |
| JY7586 | *h^-^ cut23-547 dnt1Δ::kan^R^ mad2Δ::ura4^+^ ura4- leu1-* | This study |
| JY7989 | *h^+^ cut23-547 dnt1Δ::kan^R^ mad3Δ::ura4^+^ ura4-* | This study |
| JY7463 | *h+ pDUAL-P_slp1(long2)_-slp1-T_slp1_::leu1^+^ ade6-M210 plo1-mCherry::nat^R^ cdc25-22* | [32] |
| JY8606 | *h^-^ lys1Δ::P_slp1(long2)_-slp1-T_slp1_::hyg^R^ ura4- leu1-* | This study |
| JY3060 | *h^-^ nda3-KM311 dis2Δ::ura4^+^ cdc13-117::cdc13-GFP::LEU2 ark1-as3::hyg^R^* | [25] |
| JY3600 | *h^-^ nda3-KM311 mad2Δ::ura4^+^ cdc13-117::cdc13-GFP::LEU2 ark1-as3::hyg^R^* | [25] |
| JY3542 | *h^-^ nda3-KM311 dma1Δ::ura4^+^ cdc13-117::cdc13-GFP::LEU2 ark1-as3::hyg^R^* | This study |
| JY3543 | *h? nda3-KM311 dma1Δ::ura4^+^ cdc13-117::cdc13-GFP::LEU2 ark1-as3::hyg^R^* | This study |
| JY3350 | *h^+^ da3-KM311 dnt1Δ::kan^R^ dma1Δ::ura4^+^ cdc13-117::cdc13-GFP::LEU2 ark1-as3::hyg^R^* | This study |
| JY3351 | *h^-^ nda3-KM311 dnt1Δ::kan^R^ dma1Δ::ura4^+^ cdc13-117::cdc13-GFP::LEU2 ark1-as3::hyg^R^* | This study |
| JY7803 | *h^+^ pDUAL-P_slp1(long2)_-slp1-T_slp1_::leu1^+^ ade6-210 ura4-D18* | [32] |
| JY8606 | *h^-^ lys1Δ:: P_slp1(long2)_-slp1-T_slp1_::hyg^R^ ura4- leu1-* | This study |
| JY8832 | *h^-^ pDUAL-P_slp1(long2)_-slp1-T_slp1_::leu1^+^ lys1Δ::P_slp1(long2)_-slp1-T_slp1_::hyg^R^ ura4- leu1-* | This study |
| JY8386 | *h^+^ dnt1Δ::ura4^+^ pDUAL-P_slp1(long2)_-slp1-T_slp1_::leu1^+^* | This study |
| JY8835 | *h^-^ dnt1Δ::ura4^+^ lys1Δ::P_slp1(long2)_-slp1-T_slp1_::hyg^R^* | This study |
| JY8563 | *h^+^ dnt1Δ::ura4^+^ pDUAL-P_slp1(long2)_-slp1-T_slp1_::leu1^+^ lys1Δ::P_slp1(long2)_-slp1-T_slp1_::hyg^R^* | This study |
| JY8607 | *h^+^ nda3-KM311 cdc13-117::cdc13-GFP::LEU2 pDUAL-P_slp1(long2)_-slp1-T_slp1_::leu1^+^* | This study |
| JY8731 | *h^-^ nda3-KM311 cdc13-117::cdc13-GFP::LEU2 lys1Δ::pUC119-P_slp1(1504)_-slp1-hyg^R^* | This study |
| JY8732 | *h^-^ nda3-KM311 cdc13-117::cdc13-GFP::LEU2 lys1Δ::pUC119-P_slp1(1504)_-slp1-hyg^R^* | This study |
| JY8714 | *h^+^ nda3-KM311 cdc13-117::cdc13-GFP::LEU2 pDUAL-P_slp1(long2)-_slp1-T_slp1_::leu1^+^ lys1Δ::pUC119-P_slp1(1504)_-slp1-hyg^R^ ura4-* | This study |
| JY8715 | *h^+^ nda3-KM311 cdc13-117:cdc13-GFP:LEU2 pDUAL-P_slp1(long2)-_slp1-T_slp1_::leu1^+^ lys1Δ::pUC119-P_slp1(1504)_-slp1-hyg^R^ ura4-* | This study |
| JY8112 | *h^+^ nda3-KM311 dnt1-13myc::kan^R^ mad2-13myc::hyg^R^ cut20-HA::LEU2 slp1::P_slp1_-sfGFP-slp1 leu1- ura4-* | This study |
| JY8505 | *h^-^ nda3-KM311 dnt1Δ::ura4^+^ mad2-13myc::hyg^R^ mad3-13myc::natMX6 cut20-HA::LEU2 slp1::P_slp1_-sfGFP-slp1 ade6-210 ura4-D18* | This study |
| JY8767 | *h^+^ nda3-KM311 mad3Δ::ura4^+^ dnt1-13myc::kan^R^ mad2-13myc::hyg^R^ cut20-HA::LEU2 slp1::P_slp1_-sfGFP-slp1 leu1- ura4-* | This study |
| JY8769 | *h^+^ nda3-KM311 dnt1-13myc::kan^R^ mad2-13myc::hygR mad3-13myc::natMX6* *cut20-HA::LEU2 leu1- ura4-* | This study |
| JY8770 | *h^+^ nda3-KM311 dnt1-13myc::kan^R^ mad2-13myc::hyg^R^ mad3-13myc::natMX6* *cut20-HA::LEU2 leu1- ura4-* | This study |
| JY8636 | *h^-^ cdc25-22 mad3Δ::ura4^+^ dnt1-13myc::kanR*  *mad2-13myc::hygR cut20-HA::LEU2*  *pDUAL-P_slp1(long2)_-slp1-T_slp1_::leu1^+^*  *slp1::P_slp1_-sfGFP-slp1* | This study |
| JY8667 | *h^+^ nda3-KM311 cdc13-117::cdc13-GFP::LEU2 dnt1∆::ura4^+^ pDUAL-P_slp1(long2)_-slp1-Tslp1::leu1^+^* | This study |
| JY8713 | *h^+^ nda3-KM311 cdc13-117::cdc13-GFP::LEU2 dnt1Δ::ura4^+^ pDUAL-P_slp1(long2)_-slp1-Tslp1::leu1^+^*  *lys1Δ::pUC119-P_slp1(1504)_-slp1-hygR ura4^-^* | This study |
| JY8743 | *h^+^ nda3-KM311 dnt1∆::ura4^+^ cdc13-117::cdc13-GFP::LEU2*  *lys1Δ::pUC119-P_slp1(1504)_-slp1-hygR* | This study |
| JY8892 | *h? cdc25-22 dnt1-13myc::kanR mad2-13myc::hygR cut20-HA::LEU2 pDUAL-P_slp1(long2)_-slp1-T_slp1_::leu1^+^*  *slp1::P_slp1_-sfGFP-slp1* | This study |
| JY8895 | *h? cdc25-22 dnt1-13myc::kanR mad2-13myc::hygR cut20-HA::LEU2 pDUAL-P_slp1(long2)_-slp1-Tslp1::leu1^+^* | This study |
| JY9122 | *h? nda3-KM311 apc15∆::kanR lid1-TAP-kanR mad2-GFP::kanR mad3-GFP-his3^+^* | This study |
| JY9120 | *nda3-KM311 dnt1∆::ura4^+^ apc15∆::kanR lid1-TAP-kanR mad2-GFP::kanR mad3-GFP-his3^+^* | This study |
| JY9123 | *h^-^ nda3-KM311 mad3-GFP-his3 mad2-13myc::hygR* | This study |
| JY9201 | *h? nda3-KM311 mad3-GFP-his3^+^ mad2-13myc::hygR dnt1∆::ura4^+^* | This study |
| JY9202 | *h? nda3-KM311 mad3-GFP-his3^+^ mad2-13myc::hygR apc15∆::kanR* | This study |
| JY9204 | *h? nda3-KM311 mad3-GFP-his3^+^ mad2-13myc::hygR apc15∆::kanR dnt1∆::ura4^+^* | This study |
| JY8519 | *h^+^ nda3-KM311 dnt1-13myc::kanR mad2-13myc::hygR mad3-13myc::natMX6 leu1-32 ura4-D18 ade6-210* | This study |
| JY8769 | *h^+^ nda3-KM311 dnt1-13myc::kanR mad2-13myc::hygR mad3-13myc::natMX6 cut20-HA::LEU2 leu1-32 ura4-D18* | This study |
| JY8032 | *h^-^ nda3-KM311 cut20-HA::LEU2 slp1::P_slp1_-sfGFP-slp1 leu1-32 ura4-D18* | This study |
| JY7861 | *h^+^ lys1Δ::P_adh21_-GFP::hyg^R^ dnt1Δ::kan^R^ leu1-32 ura4-D18 ade6-210* | This study |
| JY7930 | *h^+^ lys1Δ::P_adh21_-GFP-p31(comet)::hyg^R^ dnt1Δ::kan^R^ leu1-32 ura4-D18 ade6-210* | This study |
| JY7931 | *h^+^ lys1Δ::P_adh21_-GFP-2×NLS-p31(comet)::hyg^R^ dnt1Δ::kan^R^ leu1-32 ura4-D18 ade6-210* | This study |
| JY7932 | *h^+^ lys1Δ::P_adh21_-2×NLS-p31(comet)::hyg^R^ dnt1Δ::kan^R^ leu1-32 ura4-D18 ade6-210* | This study |
| JY7977 | *h^+^ lys1Δ::P_adh21_-2×NLS-p31(comet)::hyg^R^ dnt1Δ::kan^R^ leu1-32 ura4-D18 ade6-210* | This study |
| JY8005 | *h^+^ lys1Δ::P_adh21_-GFP-2×NLS-CUEDC2::hyg^R^ dnt1Δ::kan^R^ leu1-32 ura4-D18 ade6-210* | This study |
| JY8006 | *h^+^ lys1Δ::P_adh21_-2×NLS-CUEDC2::hyg^R^ dnt1Δ::kan^R^ leu1-32 ura4-D18 ade6-210* | This study |
| JY7686 | *h^+^ dnt1Δ::kanR lys1Δ::P_adh21_-dnt1::hygR leu1-32 ura4-D18 ade6-210* | This study |
| JY7862 | *h^+^ dnt1Δ::kan^R^ lys1Δ::P_adh21_-GFP-dnt1::hyg^R^ leu1-32 ura4-D18 ade6-210* | This study |
| JY8982 | *h^-^ dnt1Δ::ura4^+^ nda3-KM311 cdc13-mCherry::kan^R^ lys1Δ::P_adh21_-2×NLS-CUEDC2::hyg^R^* | This study |
| JY8979 | *h^-^ dnt1Δ::ura4^+^ nda3-KM311 cdc13-mCherry::kan^R^ lys1Δ::P_adh21_-GFP-2×NLS-CUEDC2::hyg^R^* | This study |
| JY9074 | *h^-^ nda3-KM311 cdc13-mCherry::kan^R^ dnt1Δ::kan^R^ lys1Δ::P_adh21_-GFP-dnt1::hyg^R^ leu1-32* | This study |
| JY9075 | *h^+^ nda3-KM311 cdc13-mCherry::kan^R^ dnt1Δ::kan^R^ lys1Δ::P_adh21_-GFP-dnt1::hyg^R^ leu1-32* | This study |
| JY9078 | *h^-^ nda3-KM311 cdc13-mCherry::kan^R^ dnt1Δ::kan^R^ lys1Δ::P_adh21_-dnt1::hyg^R^ leu1-32* | This study |
| JY9079 | *h^+^ nda3-KM311 cdc13-mCherry::kan^R^ dnt1Δ::kan^R^ lys1Δ::P_adh21_-dnt1::hyg^R^ leu1-32* | This study |

**Reference**

1. Guertin, D.A., Venkatram, S., Gould, K.L., McCollum, D. Dma1 prevents mitotic exit and cytokinesis by inhibiting the septation initiation network (SIN)*.* *Developmental Cell*, 2002, 3(6): 779-790.
2. Jin, Q.-W., Ray, S., Choi, S.H., McCollum, D., Bloom, K. The Nucleolar Net1/Cfi1-related Protein Dnt1 Antagonizes the Septation Initiation Network in Fission Yeast*.* *Molecular Biology of the Cell*, 2007, 18(8): 2924-2934.
3. Niwa, O., T. Matsumoto, Y. Chikashige, and M. Yanagida. 1989. Characterization of Schizosaccharomyces pombe minichromosome deletion derivatives and a functional allocation of their centromere. *EMBO J*. 8:3045-3052.
4. Takahashi, K., Yamada, H., Yanagida, M. Fission yeast minichromosome loss mutants mis cause lethal aneuploidy and replication abnormality. *Molecular biology of the cell*. 1994;5(10):1145-58.
5. Kerres, A., Jakopec, V., Beuter, C., Karig, I., Pöhlmann, J., Pidoux, A., Allshire, R., Fleig, U. Fta2, an essential fission yeast kinetochore component, interacts closely with the conserved Mal2 protein. *Molecular biology of the cell*. 2006;17(10):4167-78.
6. Chen, J.S., Lu, L.X., Ohi, M.D., Creamer, K.M., English, C., Partridge, J.F., Ohi, R., Gould, K.L. Cdk1 phosphorylation of the kinetochore protein Nsk1 prevents error-prone chromosome segregation*.* [*The Journal of Cell Biology*](http://www.baidu.com/link?url=RWq7xcRx7zNGcU5Uyb4rRRSATyCQ8lzi2RCoCHuIKpFiophCEPgl_TiyLe4rh1zY), 2011, 195(4): 583-93.
7. Hsu, K.-S., Toda, T. Ndc80 Internal Loop Interacts with Dis1/TOG to Ensure Proper Kinetochore-Spindle Attachment in Fission Yeast*. Current Biology,* 2011, 21(3): 214-220.
8. Petersen, J., Paris, J., Willer, M., Philippe, M., Hagan, I.M. The S. pombe aurora-related kinase Ark1 associates with mitotic structures in a stage dependent manner and is required for chromosome segregation*.* *Journal of Cell Science*, 2001, 114(24): 4371.
9. Liu, X., McLeod, I., Anderson, S., Yates, J.R., He, X. Molecular analysis of kinetochore architecture in fission yeast*.* *The EMBO Journal*, 2005, 24(16): 2919.
10. Sanchez-Perez, I., Renwick, S.J., Crawley, K., Karig, I., Buck, V., Meadows, J.C., Franco‐Sanchez, A., Fleig, U., Toda, T., Millar, J.B.A. The DASH complex and Klp5/Klp6 kinesin coordinate bipolar chromosome attachment in fission yeast*.* *The EMBO Journal*, 2005, 24(16): 2931.
11. Lorentz, A., Ostermann, K., Fleck, O., Schmidt, H. Switching gene swi6, involved in repression of silent mating-type loci in fission yeast, encodes a homologue of chromatin-associated proteins from Drosophila and mammals*.* *Gene*, 1994, 143(1): 139-143.
12. Partridge, J.F., Borgstrøm, B., Allshire, R.C. Distinct protein interaction domains and protein spreading in a complex centromere. *Genes Development,* 2000, 14(7):783-91
13. Rabitsch, K.P., Petronczki, M., Javerzat, J.-P., Genier, S., Chwalla, B., Schleiffer, A., Tanaka, T.U., Nasmyth, K. Kinetochore Recruitment of Two Nucleolar Proteins Is Required for Homolog Segregation in Meiosis I*.* *Developmental Cell*, 2003, 4(4): 535-548.
14. Choi, S.H., M.P. Peli-Gulli, I. McLeod, A. Sarkeshik, J.R. Yates, 3rd, V. Simanis, and D. McCollum. 2009. Phosphorylation state defines discrete roles for monopolin in chromosome attachment and spindle elongation. *Curr Biol*. 19:985-995.
15. Gregan, J., Rabitsch, P.K., Sakem, B., Csutak, O., Latypov, V., Lehmann, E., Kohli, J., Nasmyth, K. Novel genes required for meiotic chromosome segregation are identified by a high-throughput knockout screen in fission yeast*.* *Current Biology*, 2005, 15(18): 1663-1669.
16. Kawashima, S.A., T. Tsukahara, M. Langegger, S. Hauf, T.S. Kitajima, and Y. Watanabe. 2007. Shugoshin enables tension-generating attachment of kinetochores by loading Aurora to centromeres. *Genes Dev*. 21:420-435.
17. Tanaka, K., Yonekawa, T., Kawasaki, Y., Kai, M., Furuya, K., Iwasaki, M., Murakami, H., Yanagida, M., Okayama, H. Fission Yeast Eso1p Is Required for Establishing Sister Chromatid Cohesion during S Phase*.* *Molecular and Cellular Biology*, 2000, 20(10): 3459.
18. Morishita, J., Matsusaka, T., Goshima, G., Nakamura, T., Tatebe, H., Yanagida, M. Bir1/Cut17 moving from chromosome to spindle upon the loss of cohesion is required for condensation, spindle elongation and repair. *Genes to Cells*, 2001, 6(9): 743-763.
19. Trautmann, S., S. Rajagopalan, and D. McCollum. 2004. The S. pombe Cdc14-like phosphatase Clp1p regulates chromosome biorientation and interacts with Aurora kinase. *Dev Cell*. 7:755-762.
20. [Nabeshima K](https://www.ncbi.nlm.nih.gov/pubmed/?term=Nabeshima K%5BAuthor%5D&cauthor=true&cauthor_uid=7628693), [Kurooka H](https://www.ncbi.nlm.nih.gov/pubmed/?term=Kurooka H%5BAuthor%5D&cauthor=true&cauthor_uid=7628693), [Takeuchi M](https://www.ncbi.nlm.nih.gov/pubmed/?term=Takeuchi M%5BAuthor%5D&cauthor=true&cauthor_uid=7628693), [Kinoshita K](https://www.ncbi.nlm.nih.gov/pubmed/?term=Kinoshita K%5BAuthor%5D&cauthor=true&cauthor_uid=7628693), [Nakaseko Y](https://www.ncbi.nlm.nih.gov/pubmed/?term=Nakaseko Y%5BAuthor%5D&cauthor=true&cauthor_uid=7628693), [Yanagida M](https://www.ncbi.nlm.nih.gov/pubmed/?term=Yanagida M%5BAuthor%5D&cauthor=true&cauthor_uid=7628693). p93^dis1^, which is required for sister chromatid separation, is a novel microtubule and spindle pole body-associating protein phosphorylated at the Cdc2 target sites. [*Genes Dev*.](https://www.ncbi.nlm.nih.gov/pubmed/?term=p93dis1+required+for+sister+chromatid) 1995, 9(13):1572-1585.
21. Nakaseko, Y., Goshima, G., Morishita, J., Yanagida, M. M phase–specific kinetochore proteins in fission yeast: Microtubule-associating Dis1 and Mtc1 display rapid separation and segregation during anaphase. *Current Biology*, 2001, 11(8): 537-549.
22. Sato, M., Koonrugsa, N., Toda, T., Vardy, L., Tournier, S., Millar, J.B.A. Deletion of Mia1/Alp7 activates Mad2-dependent spindle assembly checkpoint in fission yeast. *Nature Cell Biology*, 2003, 5: 764.
23. Beinhauer, J.D., Hagan, I.M., Hegemann, J.H., Fleig, U. Mal3, the fission yeast homologue of the human APC-interacting protein EB-1 is required for microtubule integrity and the maintenance of cell form. *The Journal of Cell Biology*, 1997, 139(3): 717.
24. Yamashita, A., Sato, M., Fujita, A., Yamamoto, M., Toda, T. The Roles of Fission Yeast Ase1 in Mitotic Cell Division, Meiotic Nuclear Oscillation, and Cytokinesis Checkpoint Signaling*.* *Molecular Biology of the Cell*, 2005, 16(3): 1378-1395.
25. Meadows, J.C., L.A. Shepperd, V. Vanoosthuyse, T.C. Lancaster, A.M. Sochaj, G.J. Buttrick, K.G. Hardwick, and J.B. Millar. 2011. Spindle checkpoint silencing requires association of PP1 to both Spc7 and kinesin-8 motors. *Dev Cell*. 20:739-750.
26. Matsumura, T., Yuasa, T., Hayashi, T., Obara, T., Kimata, Y., Yanagida, M. A brute force postgenome approach to identify temperature-sensitive mutations that negatively interact with separase and securin plasmids*.* *Genes to Cells*, 2003, 8(4): 341-355.
27. Yamashita, Y.M., Nakaseko, Y., Kumada, K., Nakagawa, T., Yanagida, M. Fission yeast APC/cyclosome subunits, Cut20/Apc4 and Cut23/Apc8, in regulating metaphase-anaphase progression and cellular stress responses*.* *Genes to Cells*, 1999, 4(8): 445-463.
28. Hirano, T., Hiraoka, Y., Yanagida, M. A temperature-sensitive mutation of the Schizosaccharomyces pombe gene nuc2+ that encodes a nuclear scaffold-like protein blocks spindle elongation in mitotic anaphase*. The Journal of Cell Biology*, 1988, 106(4): 1171.
29. Yoon, H.-J., Feoktistova, A., Wolfe, B.A., Jennings, J.L., Link, A.J., Gould, K.L. Proteomics Analysis Identifies New Components of the Fission and Budding Yeast Anaphase-Promoting Complexes*.* *Current Biology*, 2002, 12(23): 2048-2054.
30. Yamada, H.Y., Matsumoto, S., Matsumoto, T. High dosage expression of a zinc finger protein, Grt1, suppresses a mutant of fission yeast slp1(+), a homolog of CDC20/p55CDC/Fizzy*.* *Journal of Cell Science*, 2000, 113(22): 3989.
31. Horikoshi, Y., Habu, T., Matsumoto, T. An E2 enzyme Ubc11 is required for ubiquitination of Slp1/Cdc20 and spindle checkpoint silencing in fission yeast*.* *Cell Cycle*, 2013, 12(6): 961-71.
32. Heinrich, S., Geissen, E.-M., Kamenz, J., Trautmann, S., Widmer, C., Drewe, P., Knop, M., Radde, N., Hasenauer, J., Hauf, S. Determinants of robustness in spindle assembly checkpoint signalling. *Nature Cell Biology*, 2013, 15: 1328.
