# Supplementary material for: Recovery from spindle checkpoint-mediated arrest requires a novel Dnt1-dependent APC/C activation mechanism
Source: PLoS Genet. 2022 Sep 15;18(9):e1010397. doi: 10.1371/journal.pgen.1010397 (PMC9514617; doi:10.1371/journal.pgen.1010397)
Supplement: Attachment — Submitted filename: renamed_93c87.doc [file pgen.1010397.s013.doc]

**Our point-by-point responses to three reviewers’ comments.**

**Please note that the reviewers’ comments are italicized and our responses are in blue color.**

**Reviewer #1:** This manuscript describes interesting observations showing that loss of function of the nucleolar protein Dnt1 causes a delay in anaphase inactivation of the mitotic checkpoint complex (MCC) and a consequent delay in activation of the anaphase promoting complex (APC). These effects seem to be mediated through the APC co-activator Slp1 (aka Cdc20 in other systems). The authors show reduced Slp1 levels and persistent anaphase association of Slp1 and the MCC with the APC in dnt1∆ mutant cells. While these results are interesting and in general convincing, the underlying mechanism remains unclear. The impact of the manuscript would be greatly enhanced if the authors could clearly show mechanistically whether Dnt1 is directly or indirectly involved in the phenomena described and what is the underlying mechanism.

*In addition, the manuscript is confusing and difficult to follow what the authors think their experimental results mean as you read them. It would be very helpful to build their model in the results as the manuscript progresses. For example, they should describe clearly the rationale for experiments, what hypotheses are being tested and whether the results are consistent with the hypothesis. As is, it is not always clear how the results fit into an overall model for what is going on.*

Authors’ response:

We thank the reviewer for suggestions on how to improve our writing, we have rewritten or reorganized some of the sections in our manuscript to better describe our results.

Main points:

*1) The authors want to make the case that Dnt1 directly regulates the MCC-APC. However, Dnt1 is already proposed to have a numerous other functions as well as being a component of the nucleolus. I wonder if the effects they are seeing are indirect and represent a downstream consequence to other defects in these cells.*

Authors’ response:

As the reviewer noticed, so far our previous studies have proposed Dnt1 to be involved in modulating G2/M transition (Yu *et al*., 2013; PMID: 24006256) or early mitosis progression (Wang *et al*., 2012; PMID: 22809626). So, the connection between Dnt1 and Dma1 is the only one might be relevant to Dnt1 involvement in metaphase-anaphase transition. As the reviewer suggested, we actually had suspected the delayed metaphase-anaphase transition in *dnt1∆* is the downstream consequence of Dma1 over-activation at the initial stage of this project. However, *dma1∆* only slightly rescues TBZ sensitivity of *dnt1∆* (Fig. 1A) and we did not find any evidence that Dma1 functions in regulating the abundance of Slp1^Cdc20^ (Supplementary Fig. 5) or APC/C activation (data not shown in our manuscript).

In human cells, depletion of CUEDC2 causes a checkpoint-dependent delay of the metaphase-anaphase transition, similar to that observed in *dnt1∆* mutant in *S. pombe*. Phosphorylated CUEDC2 binds to Cdc20, promotes Mad2 disassociation from APC/C^Cdc20^, thus release of APC/C from checkpoint inhibition and subsequent spindle checkpoint inactivation (as characterized in [Gao](https://pubmed.ncbi.nlm.nih.gov/?term=Gao+YF&cauthor_id=21743465) *et al*., 2011; PMID: 21743465). These mechanisms are a lot similar to that in *dnt1∆* mutant in *S. pombe*, and what's more, human CUEDC2 partially rescues the TBZ sensitivity and defective spindle checkpoint inactivation of *dnt1∆* cells (please see our Supplementary Fig. 7). These data prompted us test the hypothesis that Dnt1 has a direct regulation on the MCC-APC. Consistently, we found that Dnt1 could interact with APC/C (Fig. 5), though we could not detect the direct interaction between Dnt1 and APC/C components by yeast two-hybrid system (Fig. S6). Given the limited sequence homology between Dnt1 and CUEDC2 (Supplementary Fig. 8), we proposed that Dnt1 associates with APC/C likely through a “mediating” factor, which remains to be identified in the future studies.

Taken together, we think the APC/C activation defects in *dnt1Δ* cells we have revealed in this study are at least partially caused by loss of the direct regulation of Dnt1 on MCC-APC/C, although the possibility of indirect regulation cannot be excluded. Thus, we modified the model (Fig. 7) and discussed the possible mechanisms in the manuscript (lines 414-427).

*2) The main argument for a direct role for Dnt1 is in Figure 5 which seeks to show that Dnt1 associates with Slp1 specifically in metaphase and anaphase and not asynchronous cells. These experiments seem a bit preliminary and should be taken further. The association of Dnt1 with Slp1 is shown in checkpoint arrested (metaphase) nda3 (tubulin mutant) cells and in these cells 40 minutes after release from the arrest when they are in anaphase. The data from asynchronous cells is not shown and should be.*

Authors’ response:

The majority of APC/C subunits are permanent APC/C components, thus they are stably associated throughout the cell cycle, such as Lid1/Apc4. However, Cdc20^Slp1^ family members are synthesized only at mitosis and their protein levels peak after metaphase-anaphase transition (Heinrich *et al*., 2013; PMID: 24161933). Please see Authors’ Response Figure 1 for a good example of detected Slp1 during a synchronized cell cycle. Therefore, it is not a good idea to IP Slp1 from asynchronous cells or interphase cells.

We have also tried to detect the interaction between Dnt1 and APC/C in asynchronous/interphase cells by Lid1/Apc4 immunoprecipitation. Not surprisingly, we did not find any indication for their possible interaction during interphase. Please see Fig. 5C in our revised manuscript.

**Authors’ Response Figure 1.** Slp1 protein level fluctuates during cell cycle, with a peak after metaphase. [This image is taken from Heinrich *et al*., Nature Cell Biology, 2013; DOI: 10.1038/ncb2864] *cdc25-22* cells were cultured in rich or minimal medium, synchronized in G_2_ and released into mitosis at 16 ºC. Samples were taken every 10 min and cell extracts were analyzed for Slp1 abundance by immunoblotting with Cdc2 as the loading control. Mitotic index (MI) indicates fraction of (pro)metaphase cells at each time point.

*3) The other way the Dnt1-Slp1 association is shown is in mitotic cells generated through release from a G2 arrest (cdc25-22). However, the association is not observed in these cells unless mad3 is deleted. It is not clear why mad3∆ would increase the interaction of Dnt1 with Slp1. The authors state that their data suggests that “Dnt1 physically interacts more directly with APC/C but not MCC. Supporting this scenario, the interaction between sfGFP-Slp1 and Dnt1 was enhanced by depletion of Mad3 (Fig. 5B).” I do not understand why their data suggests this, since Mad3 associates with Slp1 both when it is bound to APC and when it is not bound to APC.*

Authors’ response:

We agree with the reviewer that Mad3 associates with Slp1 both when it is bound and not bound to APC/C, but we think when Mad3 is present, it might “titrate out” a portion of Slp1 to incorporate it into MCC. While in *mad3∆* cells, possibly more APC/C assemblies devoid of MCC are available, Dnt1 gains better opportunity to bind to APC/C.

In human cells, more abundant APC/C subunits than MCC components being co-immunoprecipitated by CUEDC2 (as shown in [Gao](https://pubmed.ncbi.nlm.nih.gov/?term=Gao+YF&cauthor_id=21743465) *et al*., 2011; PMID: 21743465), although theoretically APC/C subunits or MCC components should be pulled down with equal efficiency. This can be explained by the fact that human CUEDC2 interacts directly with Cdc20 but not MCC component Mad2.

Since we found that fission yeast Dnt1 can only be co-immunoprecipitated by APC/C subunit (Apc4/Lid1-GFP/HA or sfGFP-Slp1) but not by MCC component (Mad2- and Mad3-GFP) (see our Fig. 5), these observations made us to favor the idea that Dnt1 physically interacts more directly with APC/C but not MCC, which is very similar to human CUEDC2 on this aspect.

*4) Why not test whether Dnt1 comes down with the APC directly by Lid1/Apc4 IPs in mitosis. This would bolster the argument that it is associating with the APC and not the free MCC.*

Authors’ response:

To follow the reviewer’s suggestion, we have tested whether Dnt1 comes down with the APC/C directly by Lid1/Apc4 IPs in mitosis. We found that Dnt1 indeed also comes down weakly with Lid1/Apc4 in SAC-activated cells. We have added this new data in Fig. 5B.

*5) Extensive previous proteomics analysis on the APC has not identified Dnt1, so the observed association with Dnt1 is surprising. Also, why does Dnt1 not come down with Mad3 or Mad2, which are associated with Slp1 both on and off the APC. I wonder whether Dnt1 comes down weakly with Slp1 because it is a substrate.*

Authors’ response:

We assume that previous proteomics analyses on the APC/C in *S. pombe* failed to identify Dnt1 is due to at least three reasons:

(1) So far, large-scale APC/C purification studies mostly aimed at identifying permanent components/subunits of the APC/C (e.g. Yoon *et al.*, 2002; PMID: 12477395). Because the majority of APC/C subunits are stably associated throughout the cell cycle, so those purifications were performed with asynchronized interphase cells. However, as we have shown in the current study, Dnt1 can only be detected in immunoprecipitated APC/C from mitotic cells.

(2) In addition, even previous APC/C purifications were performed in metaphase-arrested cells, Dnt1 would likely be missed because of its paucity in immunoprecipitated complexes.

(3) Furthermore, even Dnt1 appears in the list of proteomics analyses, lack of apparent homologues in other organisms also makes Dnt1 an unlikely candidate to be picked or recognized as an APC/C-associated factor, or thought to be key for APC/C regulation.

Similarly, human CUEDC2 was detected as a APC/C-associating factor by direct co-immunoprecipitation (see [Gao](https://pubmed.ncbi.nlm.nih.gov/?term=Gao+YF&cauthor_id=21743465) *et al*., 2011; PMID: 21743465; and Authors’ Response Figure 2a, 2b), rather than being identified by proteomics analyses of APC/C, although CUEDC2 directly interacts with Cdc20 (see Authors’ Response Figure 2d).

To answer why Dnt1 does not come down with Mad3 or Mad2 (as we have shown in Fig. 5D), we think human CUEDC2 is again a good example. From data shown in previous study by [Gao](https://pubmed.ncbi.nlm.nih.gov/?term=Gao+YF&cauthor_id=21743465) *et al*. (2011; PMID: 21743465), it is clear that more Cdc27 (APC/C component) than Mad2 comes down with IPed CUEDC2 (see Authors’ Response Figure 2a, 2b). And even more strikingly, in HEK 293T cells, strong association between Cdc20 and CUEDC2, but only slight association between CUEDC2 and Mad2, could be detected by immunoprecipitation of CUEDC2 (see Authors’ Response Figure 2c). These observations support the idea that the efficiency of APC/C subunit or MCC component being co-immunoprecipitated by CUEDC2 differs significantly. We believe this difference may also apply to Dnt1, which can be co-immunoprecipitated by APC/C subunit (Apc4/Lid1-GFP/HA) but not by MCC component (Mad2- and Mad3-GFP).

Finally, we do not think Dnt1 can be a substrate of Slp1, because we have never observed changes of Dnt1 protein levels during cell cycle. We would favor the idea that Dnt1 interacts with APC/C likely through an unidentified “mediator” protein, this may explain why Dnt1 comes down only weakly with Slp1 or Apc4/Lid1.

|  | |
| --- | --- |
|  |  |
| **Authors’ Response Figure 2.** [These images are taken from Figure 4 previously shown in [Gao](https://pubmed.ncbi.nlm.nih.gov/?term=Gao+YF&cauthor_id=21743465) et al., Nature Cell Biology, 2011; PMID: 21743465; doi: [10.1038/ncb2287](https://doi.org/10.1038/ncb2287)]  (a, b) In HeLa cells, Cdc20, Mad2 and APC/C component Cdc27 were co-precipitated with CUEDC2 in nocodazole-arrested cells (a), but only Cdc20 and Cdc27, not Mad2, were still associated with CUEDC2 in cells released from the nocodazole arrest (b).  (c) In HEK 293T cells, strong association between Cdc20 and CUEDC2, but only slight association between CUEDC2 and Mad2, could be detected by immunoprecipitation of CUEDC2.  (d) They could detect a direct interaction between Cdc20 and CUEDC2 using *in vitro*-translated proteins. | |

*6) I had a hard time understanding the logic and overall conclusions in the section titled: “Enhanced MCC-APC/C association and lowered Slp1Cdc20 abundance in dnt1∆ cells can be reversed by depletion of Apc15”. The overall conclusion is well stated at the end of the section. It would be helpful to state this at the beginning by saying something like: “One possibility for Dnt1 function is…… Several lines of evidence support this model…” This would give the reader some context to make the rationale and significance of the subsequent experiments more apparent. This same critique (and suggestion) could be applied for much of the manuscript.*

Authors’ response:

We have rewritten the section as the reviewer suggested.

*7) In Figure 4C the authors state that they are looking at the amount of Slp1 associated with the MCC but not the APC by examining how much Slp1 comes down in Mad3 immunoprecipitations. But if you IP Mad3 you should get both pools of Mad3 associated with just the MCC and pools associated with the APC, so I do not understand the rationale for why Mad3 IPs only pull down Slp1 associated with the MCC and not the APC.*

Authors’ response:

We apologize for the confusion caused by our inappropriate description on our experiment. We have rewritten the relevant section in our revised manuscript. Actually, the major purpose of the experiments shown in Fig. 4A and 4B (previous Fig. 4B and 4C) is to investigate how interaction between MCC or Slp1 and APC/C in *dnt1∆* cells is affected when *apc15* is absent, which is known to disrupt MCC-APC/C association.

We agree with the reviewer that when we immunoprecipitated Mad3, we likely get both pools of Mad3 associated with just the MCC and pools associated with the APC/C. However, we reasoned that when we IPed Mad3 in the *apc15∆* background, we would detect only the MCC-associated Slp1 (via Mad3) but not APC/C-associated Slp1. As expected, more Slp1 is associated with Mad3 in *dnt1∆ apc15∆* cells compared to that in *dnt1∆* single mutant (as shown in Fig. 4B).

Additionally, in the co-IP experiments with Lid1-TAP and Mad3-GFP, we noticed that the relative abundance of Slp1 present in immunocomplexes recovered from Lid1-TAP and Mad3-GFP IPs performed in *dnt1*∆ cells was indeed very different, with much more Slp1 being co-immunoprecipitated from Lid1-TAP IP sample than from Mad3-GFP sample (Please compare the *dnt1*∆ samples with wild-type samples in Fig. 4A and 4B). This is quite surprising, and might be explained by at least two possibilities:

(1) The efficiency of Slp1 being co-immunoprecipitated by either APC/C subunit or MCC component differ significantly, although Mad3 can mediate the tight association between APC/C and MCC upon SAC activation, which should theoretically result in equal Slp1 co-immunoprecipitation capability by either complex.

(2) Larger proportion of “free” MCCs is still present within nucleoplasm compared to that of APC/C-occupied MCCs, even when SAC is robustly activated, which may cause the seemingly “dilution” out of the MCC-associated Slp1 relative to the overall APC/C and MCC- associated Slp1.

Whatever the reason is, these data suggested that the increased amount of APC/C-associated Slp1 is likely “trapped” in the interface between MCC and APC/C, which is unable to fulfill its function as the activator for APC/C in *dnt1*∆ cells.

Minor Points.

*1) Lines 217-219: States “Consistently, deletions of mad2+ or bub1+ completely abrogated this effect of dnt1∆ on delaying spindle checkpoint inactivation (Fig. 2I).” I do not think this makes sense because in these cells there is no spindle checkpoint in the first place, so you cannot look at the effect dnt1∆ on checkpoint inactivation. I think these sentences could be deleted.*

Authors’ response:

We thank the reviewer for pointing this out. We have rephrased this sentence.

*2) Lines 280-282: “……whereas the deletion of apc15^+^ abolished the MCC-APC/C interaction both in wild-type and dnt1∆ cells (Fig. 4B).” This seems to be true for Mad2 and Slp1, but not Mad3. Is this significant and has it been reported previously?*

Authors’ Response:

We thank the reviewer for pointing this out. Indeed, two previously published papers demonstrated that the level of MCC components (including Mad2 and Mad3) bound to the APC/C during mitosis is strongly reduced, but MCC assembly is intact, in *apc15∆* cells (Please see Fig. 4C and 4D in Sewart and Hauf, 2017; PMID: 28366743,), and Fig. 2A in May et al., 2017; PMID: 28366744).

To examine whether the low amount of Mad3 is reproducibly detected in immunoprecipitated APC/C complexes from *apc15∆* cells using Lid1-TAP, we repeated this experiment by including a negative control strain carrying only Mad3-GFP and Mad2-GFP but without Lid1-TAP. We noticed that low amount of Mad3-GFP but not Mad2-GFP could be detected when immunoprecipitations using IgG Sepharose beads were performed with this negative control strain (data not shown), indicating that Mad3-GFP likely binds to Sepharose beads unspecifically. By increasing the salt (NaCl) concentration from originally used 150 mM to 300 mM in our lysate buffer, we succeeded in eliminating the unspecific associations of Mad3-GFP to beads in this negative control strain and also *apc15∆* background strains, but Mad3-GFP, Mad2-GFP and Slp1 associating with Lid1-TAP persisted in wild-type and *dnt1∆* cells. Thus, our data still supports the conclusion that deletion of *apc15^+^* abolished the MCC-APC/C interaction both in wild-type and *dnt1∆* cells.

We have replaced the previous version of Fig. 4B with newly obtained images in our revised manuscript Fig. 4A, which included the negative control sample. We have also added a brief description in section “MATERIALS AND METHODS” on the change of the NaCl concentration in lysate buffer.

*3) In Figure 6, why do the authors think that dnt1∆ cells do not survive prolonged checkpoint arrest and release? If anything one might think that they would be better at surviving because they arrest better. It seems more likely that they do not decisively turn off the checkpoint and activate the APC in anaphase, leaving some securin and cyclin around and that interferes with anaphase chromosome segregation and mitotic exit.*

Authors’ response:

Honestly, we do not know the exact underlying mechanism why *dnt1∆* cells lose viability after they have experienced SAC activation. The similar phenomenon has been observed in *bub3∆* mutant, as reported in a previously published paper (Vanoosthuyse *et al*., 2009; PMID: 19846658).

One thing is clear that prolonged SAC activation generally leads to apoptosis in mammalian cells, thus is detrimental to those cells. In *S. pombe*, as an extreme example, overexpression of major components of SAC, either Mad2 or Mph1^Mps1^ causes lethality as shown previously (Please see He *et al*., 1997; PMID: 9223296 and He *et al*., 1998; PMID:9601094).

The possible mechanism proposed by this reviewer is quite plausible, but it awaits further investigation in the future.

**Reviewer #2:** In this manuscript the author has examined the role of dnt1 in the timely inactivation of spindle assembly checkpoint to efficiently initiate anaphase. These events are crucial for proper recovery of cells after APC mediated inhibition by spindle checkpoint proteins and mitotic exit. The authors have shown that the Dnt1 inhibit prolonged association of mitotic checkpoint complex with APC/C during spindle assembly checkpoint inactivation. The genetic interaction studies and expression analysis revealed that the Dnt1 positively regulates the protein level of Slp1/Cdc20 protein.

Over all the manuscript is well written and observations are informative and very interesting, there are some clarifications that I would like to be addressed before publication.

*1) Fig. 2F and G. The association of Mad2 with Lid1 in the absence of dnt1 persist longer than Mad3 association (Fig. F) but in graph (G) it is shown otherwise.*

Authors’ response:

We apologize for mislabeling the Mad2 and Mad3 in our earlier version of the figure, we have corrected this mistake in our revised figure 2G.

*2) Fig. 2A why there are two different concentration of HU was used for synchronization?*

Authors’ response:

We adopted this HU (hydroxyurea) treatment-based arrest-and-release method from one previously published paper ([Alyssa E Johnson](https://pubmed.ncbi.nlm.nih.gov/?term=Johnson+AE&cauthor_id=21131906) and [Kathleen L Gould](https://pubmed.ncbi.nlm.nih.gov/?term=Gould+KL&cauthor_id=21131906), 2011; PMID: 21131906). Cells were first arrested in S phase with HU, then released synchronously at 18 ^o^C to activate the spindle checkpoint. In this cell synchronization method, cells were treated with two doses of HU [successively](http://www.baidu.com/link?url=t9UXkvx56XqDqpIWIePW1AYrjiqsGkmnmAo-_sozlzhcBUWv3MaoWehsMBOriOLjrP3luwJ41nC7zHBp2o-H7qWoSJBH8EJRlnSmtlrC1qa) (first dose of 12 mM for 2 hours, and followed by a second dose of 6 mM for 3.5 hours). This procedure aimed to avoid the leak of the S phase arrest, thus could help to greatly improve the synchronization efficiency.

In our previous version of manuscript, we labeled the second dose of HU as 18 mM in our Fig. 2A, which actually took the first dose of HU into accounted as the final concentration. We realized that this way of labeling could mislead the readers, thus we have changed the labeling for second dose back to 6 mM in our revised Fig. 2A, in order to be consistent with our description on the method in the “Materials and Methods” section.

*3) Fig. 3A The negative genetic interaction was observed with APC subunits cut9, cut20 and cut23 mutants but not with nuc2 and apc15 mutants. Author could please explain the reason for the same.*

Authors’ response:

Actually, *dnt1∆ nuc2-663* double mutant grows slightly worse than single *nuc2-663* mutant at 33 ^o^C, demonstrating that *dnt1∆* and *nuc2-663* indeed have recognizable negative genetic interaction, though it is much weaker than other tested APC/C mutants. Whereas for *apc15∆* and *slp1-mr63* mutants, they are defective either in MCC-APC/C association (as demonstrated in Sewart and Hauf, 2017; PMID: 28366743) or in Slp1-Mad2 binding (as demonstrated in Kim *et al.*, 1998; PMID: 9461438) respectively, and thus both are defective in spindle checkpoint arrest due to removed or lessened inhibitory effect of MCC on APC/C, so it is not surprising to see the absence of their negative genetic interaction with *dnt1∆*.

*4) Fig. 3A Why slp1-362 mutant is not growing properly even at 25^o^C,*

Authors’ response:

Indeed, *slp1-362* is a rather strong temperature-sensitive (*ts*) mutant, it grows poorly even at 25 ºC. Its poor growth at 25 ºC can be revealed most apparently by spot assay as shown in our Fig. 3A, but not by being streaked on plates as shown in two previous papers: Fig. 3 in Matsumoto, 1997; PMID: 9001228; and Fig. 5A in Yamada *et al*., 2000; PMID: 11058086.

This feature was observed when *slp1-362* mutant was first isolated by Matsumoto (Molecular and Cellular Biology, 1997; PMID: 9001228). And its poor growth at 25 ºC has been documented in two aforementioned papers (Matsumoto, 1997; PMID: 9001228 and Yamada *et al*., 2000; PMID: 11058086). Please see Table 1 and Table 2 in those two papers respectively for direct comparison.

*5) Fig. 3B The nda3-KM311 synchronization strategy was not properly mentioned in figure legend like how long the cells were incubated at low temperature before releasing them at 30^o^C.*

Authors’ response:

Thanks for the reviewer’s suggestion. We have added a sentence “Strains with indicated genotypes were grown, treated and synchronized as in Fig. 2B.” in legend for Fig. 2B in our revised manuscript, which could serve as a brief description on the synchronization strategy.

*6) Fig. 3D If author has tried to express the Slp1 under nmt1 promoter to suppress the TBZ sensitivity of dnt1Δ cells. Further what could be the reason slp1 expression at leu1 locus cannot suppress the TBZ sensitivity but expression at lys1 locus suppresses well.*

Authors’ response:

(1) For the first part of the question, we have actually tried to express the Slp1 under inducible *nmt1* or constitutive *adh1* (alcohol dehydrogenase) promoter to suppress the TBZ sensitivity of *dnt1Δ* cells, but we failed to see any rescuing effect (data not shown in our manuscript). We think our result can be explained by observations reported by Silke Hauf laboratory in their Current Biology (2017) paper (PMID: 28366743). They noticed that, for some unknown reasons, expressing *slp1* from either constitutive *adh1* promoter or inducible *nmt41* promoter leads to increased Slp1 concentration in interphase but lowered concentration in mitosis (Please see Authors’ Response Figure 3). Therefore, the best way for overproducing Slp1 is through creating multiple copies of *slp1^+^* driven by its own promoter as we did in this study, especially when we need to elevate Slp1 levels at mitotic metaphase.

|  |  |
| --- | --- |
| **Authors’ Response Figure 3.** Expressing *slp1^cdc20^* from either constitutive *adh1* (alcohol dehydrogenase) promoter or inducible *nmt41* promoter leads to increased Slp1^Cdc20^ concentration in interphase but lowered concentration in mitosis.  [This image is taken from Fig. S2 in Sewart and Hauf, 2017; PMID: 28366743]  Original Fig. S2. Slp1^Cdc20^ concentration after expression from different promoters.  (A, B) Immunoblotting of extracts from cells expressing *slp1^+^* from different promoters. Cells also expressed the conditional tubulin *nda3-KM311* mutant. Samples were taken directly before (0 hrs) and 10 hrs after shifting the cultures to the restrictive temperature. An extract from cells expressing only sfGFP-tagged Slp1 was loaded as a control (*cross-reaction). In 2x *P_rad21_* strains, *slp1^+^* was expressed at the endogenous locus as well as at the exogenous *leu1* locus from the *rad21^+^* promoter. In (B), cells expressed *slp1^+^* from the regulatable *nmt41* promoter, and some samples were treated with YAM2 to suppress expression. | |

(2) For the second part of the question, we should point out that *slp1^+^* expression at both *leu1* and *lys1* loci is driven by its own promoter but followed by different terminator sequences. The leu1-32::slp1^+^ allele is flanked by *slp1* terminator sequence, whereas the lys1Δ::slp1^+^ allele is flanked by *adh1* terminator sequence. We believe these different terminator sequences caused slightly different levels of Slp1 proteins, as we have shown in Fig. 3D.

We think that the TBZ sensitivity of *dnt1Δ* cells is due to at least two aspects of defects in this mutant: one is the lowered Slp1 protein level, and the other is the enhanced and prolonged MCC-APC/C association. Although the lys1Δ::slp1^+^ allele could rescue the TBZ sensitivity of *dnt1Δ* cells slightly better than the leu1-32::slp1^+^ allele, actually simply bringing Slp1 back to or even slightly higher (e.g. in strain with 3 copies of slp1^+^) than wild type level was unable to fully suppress the TBZ sensitivity, as shown in our Fig. 3D and 3E. This failure of complete rescue is most likely due to the possibility that enhanced MCC-APC/C association persists and APC/C activity is still under tight inhibition.

**Have all data underlying the figures and results presented in the manuscript been provided?**
Large-scale datasets should be made available via a public repository as described in the *PLOS Genetics* [data availability policy](http://journals.plos.org/plosgenetics/s/data-availability), and numerical data that underlies graphs or summary statistics should be provided in spreadsheet form as supporting information.

***Reviewer #1:*** ***No:*** *Underlying numerical data not provided.*

***Reviewer #2:*** *Yes*

Authors’ response:

We have included the raw numerical data file and original images of uncropped blots as supplementary files in our revised manuscript submission.
